# *In Situ* Hybridization Analysis of the Expression of *Futsch*, *Tau*, and *MESK2* Homologues in the Brain of the European Honeybee (*Apis mellifera* L.)

**DOI:** 10.1371/journal.pone.0009213

**Published:** 2010-02-16

**Authors:** Kumi Kaneko, Sayaka Hori, Mai M. Morimoto, Takayoshi Nakaoka, Rajib Kumar Paul, Tomoko Fujiyuki, Kenichi Shirai, Akiko Wakamoto, Satomi Tsuboko, Hideaki Takeuchi, Takeo Kubo

**Affiliations:** 1 Department of Biological Sciences, Graduate School of Science, The University of Tokyo, Bunkyo-ku, Tokyo, Japan; 2 DNA Chip Research Inc., Tsurumi-ku, Yokohama, Kanagawa, Japan; Pennsylvania State University, United States of America

## Abstract

**Background:**

The importance of visual sense in Hymenopteran social behavior is suggested by the existence of a Hymenopteran insect-specific neural circuit related to visual processing and the fact that worker honeybee brain changes morphologically according to its foraging experience. To analyze molecular and neural bases that underlie the visual abilities of the honeybees, we used a cDNA microarray to search for gene(s) expressed in a neural cell-type preferential manner in a visual center of the honeybee brain, the optic lobes (OLs).

**Methodology/Principal Findings:**

Expression analysis of candidate genes using *in situ* hybridization revealed two genes expressed in a neural cell-type preferential manner in the OLs. One is a homologue of *Drosophila futsch*, which encodes a microtubule-associated protein and is preferentially expressed in the monopolar cells in the lamina of the OLs. The gene for another microtubule-associated protein, *tau*, which functionally overlaps with *futsch*, was also preferentially expressed in the monopolar cells, strongly suggesting the functional importance of these two microtubule-associated proteins in monopolar cells. The other gene encoded a homologue of Misexpression Suppressor of Dominant-negative Kinase Suppressor of Ras 2 (MESK2), which might activate Ras/MAPK-signaling in *Drosophila*. *MESK2* was expressed preferentially in a subclass of neurons located in the ventral region between the lamina and medulla neuropil in the OLs, suggesting that this subclass is a novel OL neuron type characterized by *MESK2*-expression. These three genes exhibited similar expression patterns in the worker, drone, and queen brains, suggesting that they function similarly irrespective of the honeybee sex or caste.

**Conclusions:**

Here we identified genes that are expressed in a monopolar cell (*Amfutsch* and *Amtau*) or ventral medulla-preferential manner (*AmMESK2*) in insect OLs. These genes may aid in visualizing neurites of monopolar cells and ventral medulla cells, as well as in analyzing the function of these neurons.

## Introduction

Some insect species possess a highly developed visual sense that is essential for adaptation to the environment [1], [2]. The European honeybee (*Apis mellifera* L.) is a social insect, and its colony consists of three types of adults: queens (female reproductive caste), workers (female labor caste), and drones (reproductive males) [3]. In addition, the workers shift their labors from nursing their brood (nurse bees) to foraging (foragers), according to their age after adult emergence [3]. Highly developed visual ability is especially important for social Hymenopteran insects, because they must be able to return to their hive [1], [2]. For example, queens and drones mate in the air several tens of meters above the ground (rendezvous flight), which might require visual memory to return to the hive [3]. Workers use various visual cues to memorize the locations of food sources and return to the hive from several kilometers away [3]. In particular, they use optic flow to gauge the distance from the hive to the food source and inform their nestmates of the location of the food source (distance and direction from the hive) using the well-known ‘dance communication’ [3]–[6].

In the honeybee, visual information perceived at the compound eyes is first projected to the optic lobes (OLs), a visual center of the insect brain, and is then projected to other brain areas, such as the mushroom bodies (MBs), a higher-order integration center of the insect brain [7]–[11]. The OLs are composed of three layers of neuropil: the lamina, which directly contacts the retina; the medulla; and the lobula [7]. Electrophysiologic studies suggest that the proportion of neurons involved in the detection of light wavelength, location, and motion direction differs in each of the three neuropil layers [12]–[19]. Anatomically, neurons in the OLs comprise groups termed ‘cartridges’ in the lamina, and groups termed ‘columns’ in the medulla and lobula. Neurons in these cartridges and columns are classified into subtypes based on their morphology or projection patterns [7], [20]–[23]. The ability of honeybees to discriminate various colors, shapes, patterns, and motion direction have been studied in both free-flying [4], [24] and harnessed bees [25]–[27]. Due to the small and rather simple honeybee brain and their high visual abilities, the honeybee represents an excellent model for analyzing visual information processing in the brain.

The importance of visual ability in the honeybee might be reflected in their brain structures. The MB structure changes depending on the division of labor of the workers from nursing to foraging, and correlates with the foraging experience of the foragers [28], [29]. It is thus plausible that visual experience affects patterns or densities of axonal projections of OL neurons to the MBs. In addition, in Hymenopteran insects, visual information processed in the OLs is projected directly to the MBs [7], [9], [30], [31], whereas in many other insect species, such as the fruit fly, the MBs are important for olfactory processing and there are few or no direct neuronal connections between the OLs and MBs [7], [10], [32]. Some OL cell types might be responsible for gauging the optic flow, which would help to make foraging behavior possible. In addition, OL neurons might have specific neural plasticity that underlies the changes in the MB structure depending on the division of labor and/or foraging experiences. The molecular and neural bases that underlie the honeybee visual abilities essential for their social behaviors, however, remain largely unknown.

Recent studies revealed that many genes are expressed in a MB-preferential manner in the adult honeybee brain [33]–[40], suggesting that functional specialization of the MB depends on the distinct gene expression profiles. Functional analysis of these genes by manipulating their expression using reverse genetic methods will provide important clues for understanding the MB-dependent brain functions. In addition, these genes might be used as reporter genes to aid in visualizing the MB neural circuits that express the genes by introducing fluorescent protein genes ligated downstream of their promoters, a method that has been applied in other animal species [41], [42]. Therefore, ‘molecular dissection’, in which genes expressed in a brain region-preferential manner are systematically identified, could be a promising methodology for analyzing the molecular and neural bases of honeybee brain functions. Although transcriptomic profiling of central nervous system regions in three honeybee species has also be reported [43], specific gene(s) that may be useful markers for labeling the OL neural circuits in insect brains have not been identified in insects, including honeybees.

The present study aimed to identify gene(s) with various expression patterns in the OLs in the honeybee brain. A cDNA microarray was used to screen genes that are expressed strongly in the OLs in the honeybee brain, and then expression analyses of the candidate genes were performed to reveal their expression patterns in the OLs. Here, we report the identification of two genes expressed in a neural cell type-preferential manner in the OLs of the honeybee: The first was a homologue of *Drosophila futsch*, which encodes a microtubule-associated protein [44], [45], and is preferentially expressed in the monopolar cells in the lamina of the OLs. In addition, the gene for another microtubule-associated protein, *tau* [46], [47], was also preferentially expressed in the monopolar cells. The second gene encoded a homologue of Misexpression Suppressor of Dominant-negative Kinase Suppressor of Ras 2 (MESK2) [48]. These genes exhibited similar expression patterns irrespective of the sex or caste of the honeybees.

## Results

### Gene Structure of Amfutsch, Which Was Identified by Screening of Genes Strongly Expressed in the OLs in the Honeybee Brain

To obtain useful marker genes for labeling specific neurons in the honeybee OLs, we first searched for genes that were expressed more strongly in the OLs than in the other brain regions, based on the supposition that such genes would be more suitable for discriminating OL neurons that express the genes. We used a cDNA microarray with over 5000 cDNA subclones that represent various genes expressed in the adult honeybee brains to screen gene(s) that are expressed more strongly in the OLs than in the other regions of the honeybee brain (for details of the screening, see the Materials and Methods section) [35], [39]. *In situ* hybridization of the candidate clones led to the identification of two clones (Clone #1 and #2).

**Figure 1:**
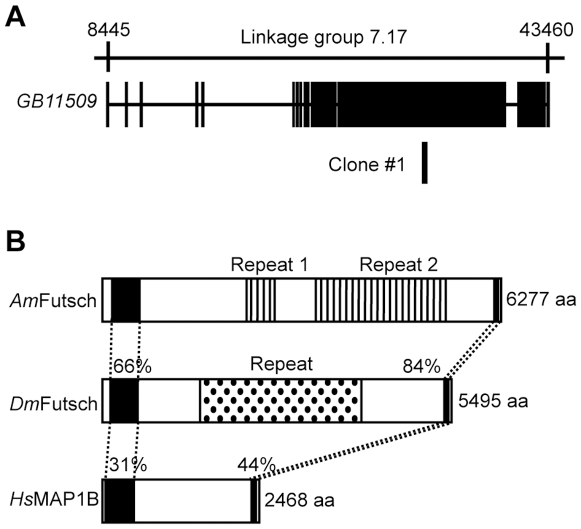
Gene structure of the predicted gene corresponding to Clone #1. Genomic organization of the predicted gene corresponding to Clone #1(**A**) and comparison of the domain structure of Futsch/MAP1B (**B**). (**A**) Exon (closed boxes) and intron (lines) structures of the predicted gene and the location of Clone #1 are indicated below the corresponding linkage group (upper line). (**B**) The two closed boxes in *Am*Futsch, *Dm*Futsch and *Hs*MAP1B indicate the N-terminal and C-terminal conserved regions, respectively. The two striped boxes in *Am*Futsch and the dotted box in *Dm*Futsch indicate tandem Repeat 1 and 2, and Repeat, respectively. The numbers above the conserved regions indicate sequence identities with *Am*Futsch.

An NCBI database (http://www.ncbi.nlm.nih.gov/) search revealed that Clone #1 corresponded to a part of the exon of a predicted honeybee gene, *GB11509*, which is located at Linkage group 7 in the honeybee genome and spans approximately 35 kbp (Fig. 1A). *GB11509* [honeybee (*Am*) *futsch*] encodes a homologue of *Drosophila* Futsch (*Dm*Futsch) and a mammalian Microtubule Associated Protein 1 (MAP1), such as human MAP1B (*Hs*MAP1B). These two proteins form a protein family: a class of microtubule-binding proteins with conserved structures [44], [45]. *Dm*Futsch and *Hs*MAP1B have two highly conserved domains in both the N- and C-terminal regions, and their sequence identities with honeybee Futsch (*Am*Futsch) were 66% and 31% (N-terminal region), and 84% and 44% (C-terminal region), respectively (Fig. 1B). The proteins had distinct intervening amino acid sequences between the highly conserved domains. *Dm*Futsch contained a 66x tandem repeat of approximately 37 amino acids, SVAEKSPLASKEASRPASVAESVKDEAEKSKEESRRE [44], whereas *Am*Futsch contained two kinds of repeats in the intervening region (Fig. 1B; Repeat 1 and 2, respectively). Both sequences differed from those of *Dm*Futsch: Repeat 1 represented an approximately 25x tandem repeat of 18 amino acid sequences, KKEEKKPV/EEEEKEL/IKVEE, whereas Repeat 2 represented an approximately 50x tandem repeat of 42 amino acids, EKSRSPSVTSVTAETKEPSDKSKSPSVAGEV/KPELKDVDTKEI, where the highly frequent amino acid residues are aligned, respectively (Fig. 1B). A database search revealed that *Am*Futsch had the highest sequence similarity with *Dm*Futsch, and there was no other complete gene that had sequence similarities with *Dm*Futsch. Thus, we concluded that Clone #1 corresponded to *Amfutsch*. Clone #1 was located at the most N-terminal of Repeat 2 (Fig. 1B).

### Amfutsch Expression Analysis in the Honeybee Brain

**Figure 2:**
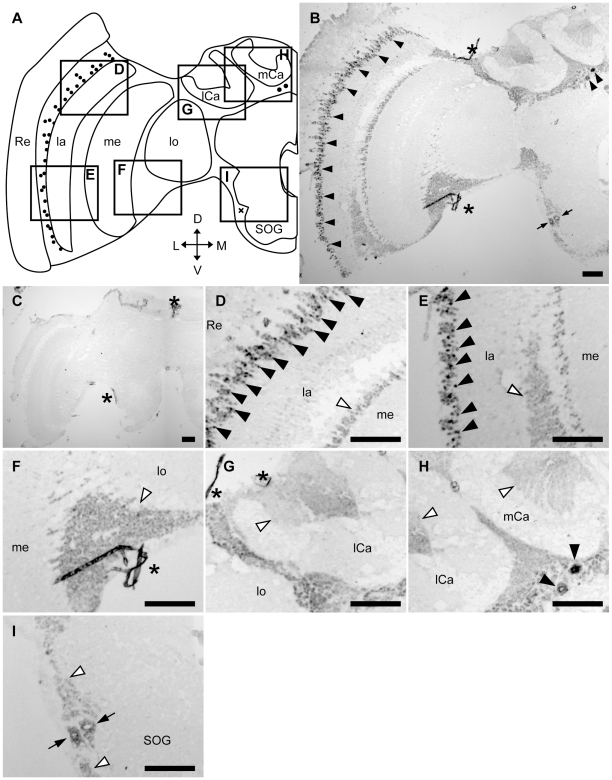
*In situ* hybridization of *Amfutsch* in the nurse bee brain. *In situ* hybridization using DIG-labeled RNA antisense (**B, D–I**) and sense (**C**) *Amfutsch* probes with nurse bee brain sections. (**A**) Schematic representation of the signals detected in the left-brain hemisphere of the nurse bee brain. Black circles and a black check mark indicate stronger or intermediate signals, respectively. (**D–I**) Magnified views of parts of (**B**) corresponding to the boxes shown in (**A**). The stronger signals detected in the lamina (**D, E**) and in the other region (**H**) are indicated by black arrowheads. White arrowheads indicate the regions with no significant signals (**D–I**). Black arrows indicated intermediate signals near the SOG (**I**). Scale bars = 100 µm. Asterisks indicate non-specific staining. D, dorsal; L, lateral; la, lamina; lCa, lateral calyx; lo, lobula; M, medial; me, medulla; mCa, medial calyx; Re, retina; SOG, subesophageal ganglion; V, ventral. Note that each panel (panels D–I) shows repeated views of the same section rather than multiple sections from multiple brains.

*In situ* hybridization using nurse bee, forager, queen and drone brain sections and *Amfutsch*-specific antisense RNA probes revealed a similar expression pattern in the brain between all of the above four bee types. In nurse bee brains, although weak signals were detected in the whole brain cortex (Fig. 2A, B), strong signals were detected in two restricted brain regions: 1) a part of the lamina cells in the OLs (black arrowheads in Fig. 2D, E); and 2) a few cells with large somata located beneath the calyces of the MBs (black arrowheads in Fig. 2H). In addition, intermediate signals were sometimes detected in two other brain regions: 1) a few cells with large somata located in the lateral area of the subesophageal ganglion (SOG; arrows in Fig. 2I); and 2) some cells located between the OLs and MBs (arrows in Figs. S2G and S3F, for queen and drone brains, respectively). Frequent, but not constant, detection of these two signals may depend on the individual experiment or the depths of the sections used. In contrast, there were no intense signals in the other brain regions, including the inside of the calyces of the MBs (white arrowheads in Fig. 2G, H), cells between the lamina and medulla (white arrowheads in Fig. 2D, E), cells between the medulla and lobula (white arrowhead in Fig. 2F), or cells located around SOG (white arrowheads in Fig. 2I). These signals were not detected in sections hybridized with sense probe (Fig. 2C), indicating that the signals were due to the expression of *Amfutsch*. Although *Amfutsch* was expressed weakly in the whole brain cortex, it was expressed preferentially in restricted neural cell types, suggesting the importance of this gene product in these cells (Fig. 2B, C). Essentially the same expression profile was detected in all forager (Fig. S1), queen (Fig. S2) and drone brains (Fig. S3), suggesting that *Amfutsch* functions similarly in these neural cell types in the honeybee brain, irrespective of the sex, caste, and worker age-polyethism.

### Analysis of Co-Expression of Amfutsch and Amtau in the OLs of the Honeybee Brain

**Figure 3:**
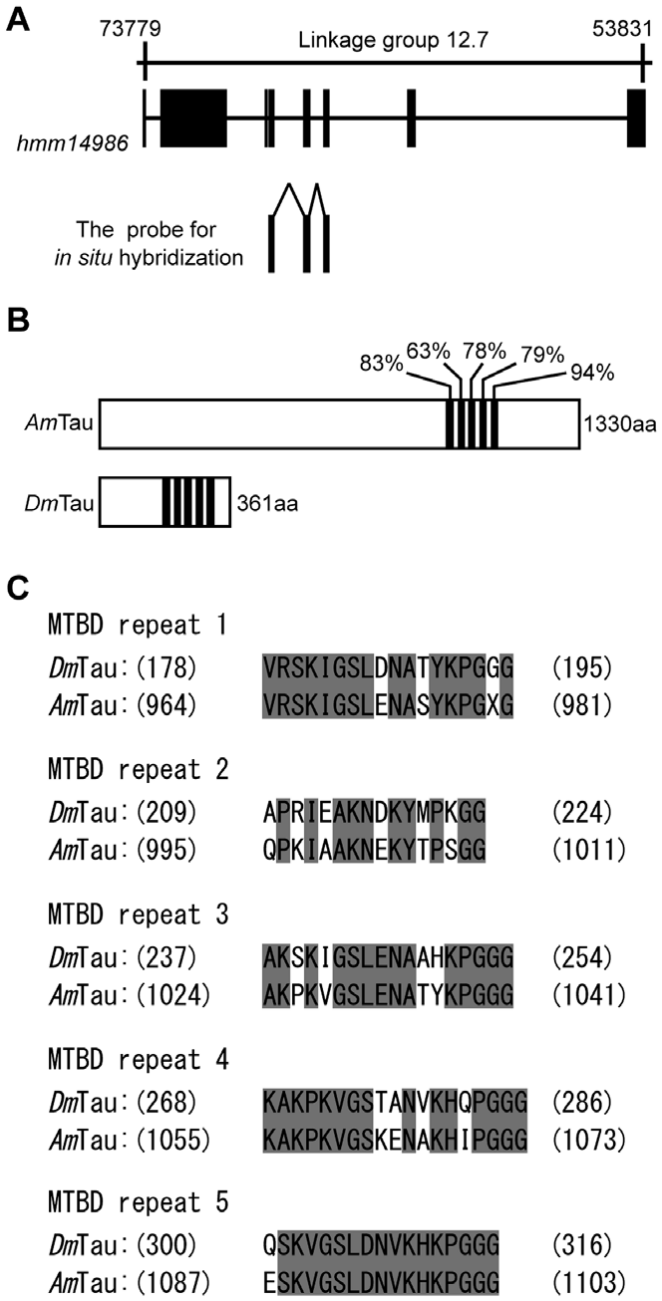
Gene structure of the predicted gene *Amtau*. Genomic organization of the predicted gene *Amtau* (**A**), comparison of the domain structures of Tau (**B**) and alignment of amino acid sequences of MTBD repeats (**C**). (**A**) Exon (closed boxes) and intron (lines) structures of the deduced gene and the location of the probe used for *in situ* hybridization are indicated below the corresponding linkage group (upper line). Closed boxes in (**B**) indicate microtubule-binding regions of *Am*Tau and *Dm*Tau. The numbers above these regions of *Am*Tau indicate sequence identities with corresponding regions in *Dm*Tau. (**C**) The amino acid sequences of MTBD Repeat 1–5 of *Dm*Tau (upper amino acid sequence) and *Am*Tau (lower amino acid sequence) are aligned, where the gray background indicate the identical amino acid residues. The numbers on the left and right of each sequence indicate amino acid positions in each protein.

We next examined the possible co-expression of *Amfutsch* with honeybee *tau* (*Amtau*) in the lamina. The gene *tau* also encodes a microtubule-associated protein, which functionally overlaps with Futsch/MAP1, and Tau works cooperatively with Futsch/MAP1 in the neurons of various animal species [44]–[47]. Therefore, we hypothesized that *Amfutsch* and *Amtau* are co-expressed in the honeybee brain. To test this possibility, we first searched for *tau* homologue in the honeybee genome using the NCBI database. Two predicted genes, *hmm14986* and *hmm75911* were identified. *Hmm14986* is located at the Linkage group 12 (Fig. 3A) and encodes a protein with five microtubule-binding domain (MTBD) repeats, each of which have high (83, 63, 78, 79 and 94%, respectively) sequence identities with the corresponding MTBD repeats of *Drosophila* Tau (*Dm*Tau) (Fig. 3B, C). On the other hand, *hmm75911* is located at the genomic contig, Un.3121, and encodes a partial amino acid sequence with three MTBD repeats (data not shown). These two genes have almost (approximately 99%) the same nucleotide sequences, which may indicate that they represent the same gene.

**Figure 4:**
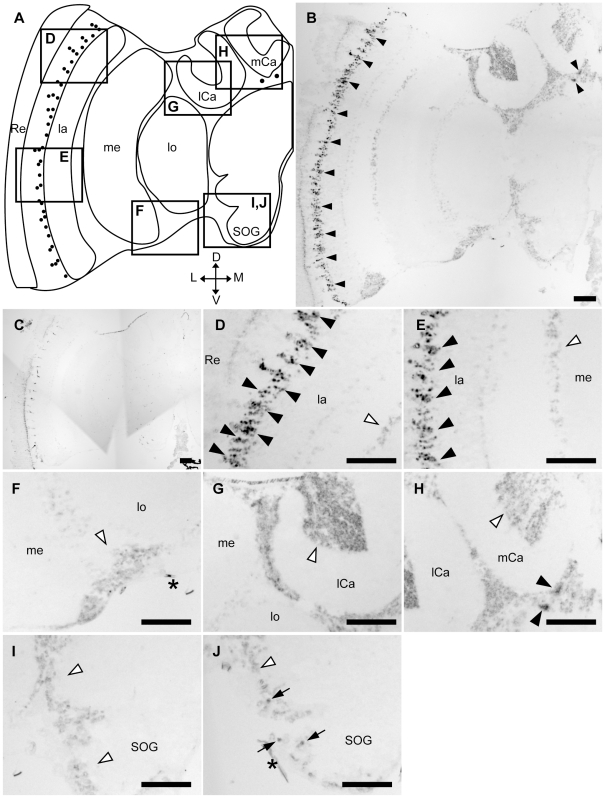
*In situ* hybridization of *Amtau* in the forager brain. *In situ* hybridization using DIG-labeled RNA antisense (**B, D–I**) sense (**C**) *Amtau* probes and the forager brain sections. (**A**) Schematic representation of signals detected in the left-brain hemisphere of the forager brain. Black circles indicate stronger signals. (**D–I**) Magnified views of parts of (**B**) corresponding to the boxes shown in (**A**). (**J**) Magnified view of the same part as (**I**) of another section, which includes intermediate signals. The stronger signals detected in the lamina (**D and E**) and the other region (**H**) are indicated by black arrowheads. White arrowheads indicate regions with no significant signals (**D–J**). Black arrows indicated intermediate signals near the SOG (**J**). Scale bars = 100 µm. Asterisks indicate non-specific staining. D, dorsal; L, lateral; la, lamina; lCa, lateral calyx; lo, lobula; M, medial; me, medulla; mCa, medial calyx; Re, retina; SOG, subesophageal ganglion; V, ventral.

In *Drosophila*, although Tau-immunoreactive cells were detected in the adult photoreceptor neurons [46], *in situ* hybridization of *Dmtau* in the adult brain has not been performed. We analyzed *Amtau*-expression in the honeybee brain by *in situ* hybridization using brain sections of foragers and nurse bees and a probe that corresponds to a region including the most N-terminal MTBD repeat of the predicted *Am*Tau (Fig. 3A). Essentially the same *Amtau*-expression profile was observed in both nurse bee (Fig. S4) and forager brains (Fig. 4). Furthermore, *Amtau*-expression in the honeybee brain resembled that of *Amfutsch*. Although weak signals were detected in the whole brain cortex (Fig. 4A, B), stronger signals were detected in two restricted brain regions in the forager brain: 1) in a part of the lamina cells (black arrowheads in Fig. 4D, E); and 2) a few cells with large somata and are located beneath the MBs (black arrowheads in Fig. 4H). In addition, intermediate signals were sometimes detected in two other brain regions: 1) a few cells with large somata located in the lateral area of the SOG (arrows in Fig. 4J); and 2) some cells located between the OLs and MBs (data not shown). In contrast, there were no intense signals in other brain regions (white arrowheads in Fig. 4D–J). These signals were not detected in sections hybridized with sense probe (Fig. 4C), indicating that the signals represented *Amtau*-expression. These results indicated that *Amfutsch* and *Amtau* were expressed in the similar brain regions. Similar to *Amfutsch*, although *Amtau* is expressed weakly in the whole brain cortex, it is expressed preferentially in restricted neural cell types, suggesting the importance of this gene product in these cells (Fig. 4B, C).

**Figure 5:**
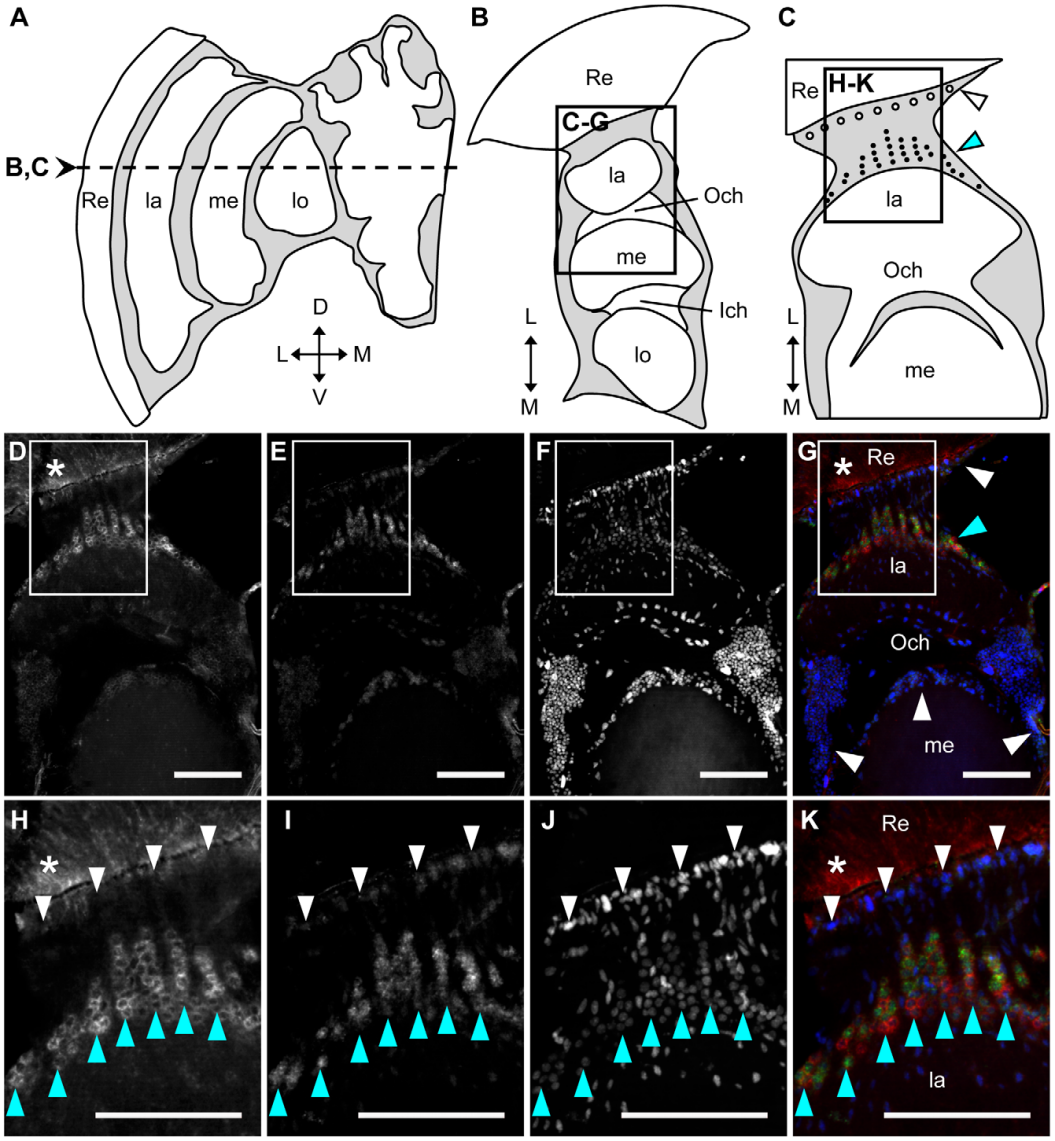
Double *in situ* hybridization of *Amfutsch* and *Amtau* in the OLs of the forager brain. Fluorescent double *in situ* hybridization using DIG-labeled RNA antisense *Amfutsch* and *Amtau* probe and horizontal sections of forager brains, followed by DAPI staining. (**A**) Schematic representation of the structure in the vertical brain sections. White regions correspond to neuropil, whereas gray regions indicate cortex. (**B**) Schematic representation of the structure in the horizontal brain section corresponding to the dotted line in (**A**). (**C**) Schematic representation of the structure in the horizontal brain section corresponding to the box shown in (**B**). Black circles indicate monopolar cells with signals, and white circles indicate glial cells with no signals. (**D–G**) *In situ* hybridization of the box shown in (**B**): (**D**) with antisense *Amfutsch* probe, (**E**) with antisense *Amtau* probe, (**F**) nuclear staining with DAPI, and (**G**) merged images in (**D–F**). Red, green, and blue signals in (**G**) indicate *Amfutsch*- and *Amtau*-expression and nuclear staining with DAPI, respectively. (**H–K**) Magnified views corresponding to the boxes shown in (**D–G**), respectively. The stronger signals detected in the lamina monopolar cells are indicated by blue arrowheads, and regions with no significant signals are indicated by white arrowheads. Scale bars = 100 µm. Asterisks indicate non-specific staining. D, dorsal; Ich, inner chiasma; L, lateral; la, lamina; lo, lobula; M, medial; me, medulla; MB, mushroom body; Och, outer chiasma; Re, retina; V, ventral.

Next, to identify the cell types that express *Amfutsch* and *Amtau* as well as to further confirm the co-expression of *Amfutsch* and *Amtau* in the lamina, we performed double fluorescent *in situ* hybridization using *Amfutsch*- and *Amtau*-specific antisense RNA probes and horizontal sections of the forager brain, followed by nuclear staining with DAPI (Fig. 5). Expression of both *Amfutsch* and *Amtau* was detected preferentially in a subclass of lamina cells located between the retina and lamina (blue arrowheads in Fig. 5D–G). The neurons and glial cells exhibit unique distribution patterns in the lamina of the OLs [20]. Comparison of the *Amfutsch/Amtau*-expressing cells by staining the nuclei with DAPI suggested that these cells were monopolar cells (blue arrowheads in Fig. 5H–K) and not glial cells (white arrowheads in Fig. 5H–K), whose cell bodies are located at the inner and outer parts of the cortex between the retina and lamina, respectively [20], although we could not definitely conclude that they are neurons but not glial cells, without staining with glial markers. The signals for both *Amfutsch*- and *Amtau*-expression were detected in monopolar cells, and they overlapped at least in some monopolar cells (Fig. 5H–K), indicating that lamina monopolar cells preferentially expressed both *Amfutsch* and *Amtau* in the OLs.

To obtain evidence that *Am*Futsch and *Am*Tau also function as ‘microtubule-associated proteins’ in the honeybee brain, we used *in situ* hybridization to examine the expression of *Amfutsch* and *Amtau* in the developing pupal brains, based on the assumption that these genes are expressed in the differentiating neurons in the developing pupal brains. As expected, both *Amfutsch* and *Amtau* were expressed in the developing pupal brains (Supporting Information S1, Fig. S5 and S6). Unexpectedly, however, these genes were differentially expressed in the pupal brains: *Amfutsch* was expressed in the whole brain cortex except for the mushroom bodies (MBs), whereas *Amtau* was expressed around the proliferating MB cells, suggesting that these genes function in a brain-region dependent manner in developing pupal brains (Supporting Information S1, Fig. S5 and S6). We could not identify differentiating monopolar cells and thus examine the expression of these genes in these cells.

### Gene Structure of Honeybee MESK2

**Figure 6:**
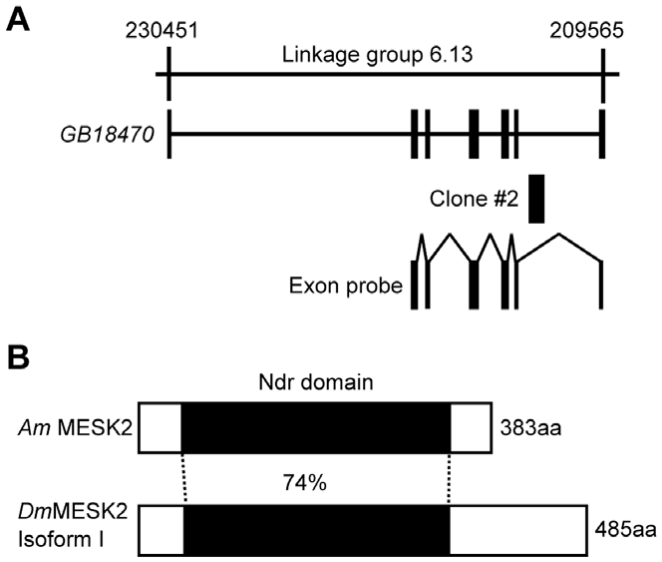
Gene structure of the predicted gene *AmMESK2*. Genomic organization of the predicted gene *AmMESK2* (**A**) and comparison of the domain structures of *Am*MESK2 and *Dm*MESK2 (**B**). (**A**) Exon (closed boxes) and intron (lines) structures of the predicted gene and the location of the Clone #2 and the probe for *in situ* hybridization are indicated below the corresponding linkage group (upperline). (**B**) Closed boxes in *Am*MESK2 and *Dm*MESK2 isoform I indicate Ndr domains. The number below the Ndr domain of *Am*MESk2 indicates sequence identities with that of *Dm*MESK2.

Clone #2 corresponded to the putative intron region of a deduced gene, *GB18470*, which is located at Linkage group 6 (Fig. 6A). NCBI database search revealed that *GB18470* encodes a protein that has the highest sequence similarity with *Drosophila* Misexpression Suppressor of dominant-negative KSR (Kinase Suppressor of Ras) 2 (MESK2). The *Drosophila* MESK2 isoform I (GenBank accession No. AAS64904.1) consists of 485 amino acid residues and contains an Ndr domain, which is the domain conserved among proteins encoded by the *N-myc downstream regulated* gene family (Fig. 6B) [48] (http://www.ncbi.nlm.nih.gov/Structure/cdd/wrpsb.cgi). The predicted protein encoded by *GB18470* consists of 383 amino acid residues and contains an Ndr domain that also has sequence identity (74%) with that of *Dm*MESK2 (Fig. 6B). Because there were no genes other than *GB18470* similar to *DmMESK2* in the honeybee genome, and the protein encoded by *GB18470* had the highest sequence similarity with *Dm*MESK2 in the honeybee genome, we concluded that *GB18470* is the *DmMESK2* homologue (*AmMESK2*).

To confirm that Clone #2 actually corresponded to the intron region of *AmMESK2*, we intended to examine whether Clone #2 is connected with the predicted 6th exon on a precursor *AmMESK2* mRNA, by amplifying partial cDNA that contained both Clone #2 and the predicted 6th exon region of *AmMESK2* by reverse transcription-polymerase chain reaction (RT-PCR) (Supporting Information S1, Fig. S7). A cDNA fragment of approximately 700 bp, which is consistent with the predicted size (678 bp), was obtained, supporting our notion that Clone #2 corresponds to an intron of *AmMESK2*. We can't exclude the possibility, however, that there are some alternative splice variants and that Clone #2 partly includes sequences for these splice variants.

### Expression Analysis of AmMESK2 in the Honeybee Brain

**Figure 7:**
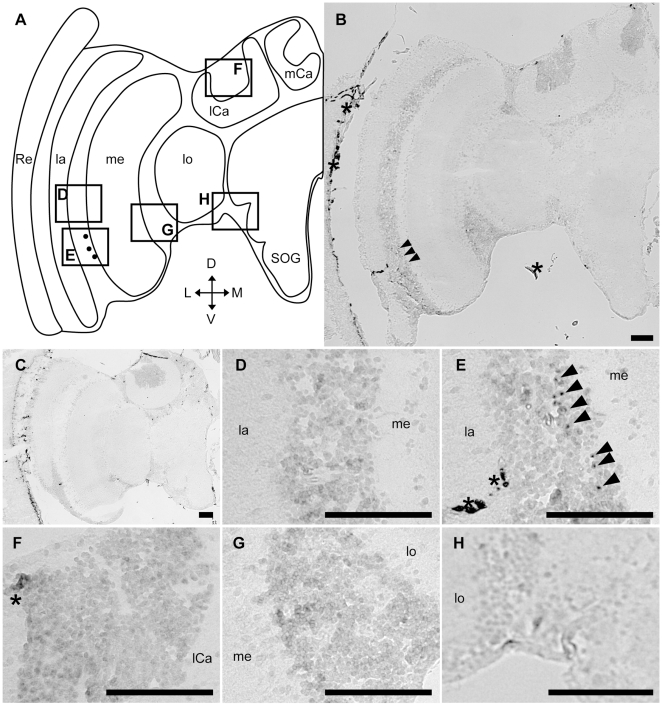
*In situ* hybridization with the intron probe of *AmMESK2* in the forager brain. *In situ* hybridization using DIG-labeled RNA antisense (**B, D–H**) and sense (**C**) *AmMESK2* probes with forager brain sections. (**A**) Schematic representation of signals detected in the left-brain hemisphere of the forager brain. Black circles indicate the stronger signals. (**D–H**) Magnified views of parts of (**B**) corresponding to the boxes shown in (**A**). The signals detected in the cortex between the lamina and medulla are indicated by black arrowheads. Scale bars = 100 µm. Asterisks indicate non-specific staining. D, dorsal; L, lateral; la, lamina; lCa, lateral calyx; lo, lobula; M, medial; me, medulla; mCa, medial calyx; Re, retina; SOG, subesophageal ganglion; V, ventral. Note that stronger signals detected in magnified view (**E**) are scarcely detected in low magnification micrograph (**B**), and so the signals need to be examined closely with magnified views. This is also the case for Figs. 8, 9, S5, S6 and S7.

*Dm*MESK2 was originally identified by screening genes with the potential to modulate RAS-signaling when misexpressed [49], and its actual functions and expression profile have not been analyzed. Therefore, we performed *in situ* hybridization of *AmMESK2* using the forager brain sections and an ‘intron probe’ that corresponded to the sequence (Clone #2) obtained by the cDNA microarray analysis. Significant signals were detected in only a single brain region: a few dozen neurons whose somata were located at the ventral part of the cortex between the lamina and medulla of the OLs (Fig. 7A, B, E). No significant signals were detected in the other brain regions including the OL (Fig. 7D, G), the inside of the calyces of the MBs (Fig. 7F), and cells around the SOG (Fig. 7H). These signals were not detected in sections hybridized with sense probe (Fig. 7C), indicating that the signals represented *AmMESK2*-expression. Interestingly, *AmMESK2* was not expressed in the dorsal or middle part of the OLs (Fig. 7D), indicating that *AmMESK* 2 was expressed preferentially in the ventral part of the OLs (Fig. 7E). Essentially the same expression profiles were detected with nurse bee brain sections (Fig. S8).

**Figure 8:**
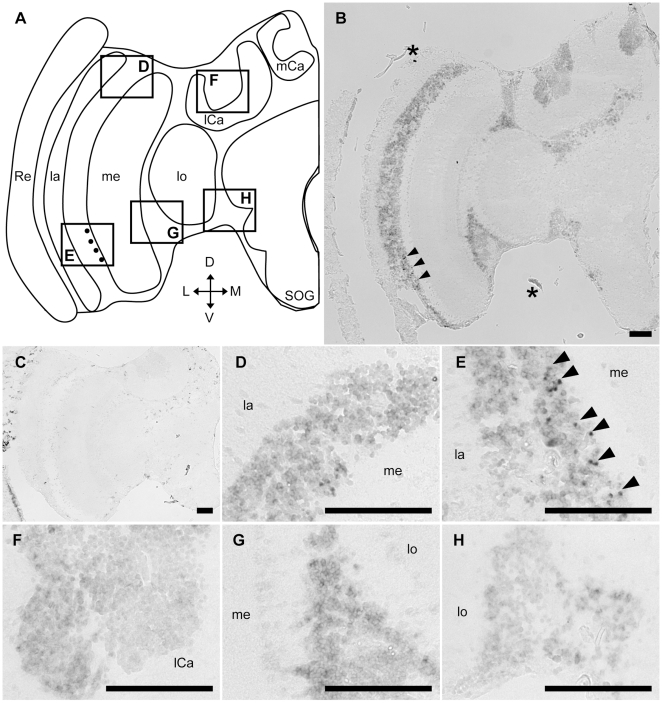
*In situ* hybridization with the exon probe of *AmMESK2* in the forager brain. *In situ* hybridization using DIG-labeled RNA antisense (**B, D–H**) and sense (**C**) *AmMESK2* probes with forager brain sections. (**A**) Schematic representation of signals detected in the left-brain hemisphere of the forager brain. Black circles indicate the stronger signals. (**D–H**) Magnified views of parts of (**B**) corresponding to the boxes shown in (**A**). The stronger signals detected between the lamina and medulla are indicated by black arrowheads. Scale bars = 100 µm. Asterisks indicate non-specific staining. D, dorsal; L, lateral; la, lamina; lCa, lateral calyx; lo, lobula; M, medial; me, medulla; mCa, medial calyx; Re, retina; SOG, subesophageal ganglion; V, ventral.

**Figure 9:**
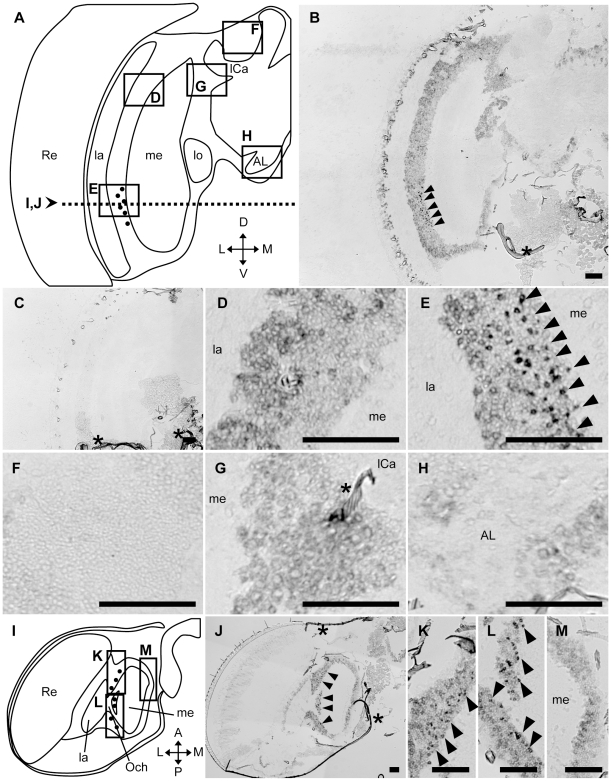
*In situ* hybridization with the exon probe of *AmMESK2* in the drone brain. *In situ* hybridization using DIG-labeled RNA antisense (vertical sections; **B, D–H**, horizontal sections; **J–M**) and sense (vertical sections; **C**) *AmMESK2* probes with drone brain sections. (**A, I**) Schematic representation of signals detected in the vertical (**A**) and horizontal section (**I**) of the left-brain hemisphere of drones, respectively. Black circles indicate stronger signals. The dotted line in (**A**) indicates the position of the horizontal sections for (**I–J**). (**D–H**) Magnified views of parts of (**B**) corresponding to the boxes shown in (**A**). (**K–M**) Magnified views of parts of (**J**) corresponding to the boxes shown in (**I**). The stronger signals detected in the cortex between the lamina and medulla are indicated by black arrowheads. Scale bars = 100 µm. Asterisks indicate non-specific staining. A, anterior; AL, antennal lobe; D, dorsal; L, lateral; la, lamina; lCa, lateral calyx; lo, lobula; M, medial; me, medulla; mCa, medial calyx; Och, outer chiasma; P, posterior; Re, retina; V, ventral.

We then analyzed *AmMESK2*-expression by *in situ* hybridization using an ‘exon probe’ designed to correspond to most of the putative exon regions, including those coding the Ndr domain, based on the supposition that stronger expression can be detected with the ‘exon probe’. The results revealed that, although weak signals were detected in most brain cortex, stronger signals were detected in the same ventral regions between the lamina and medulla in the OLs in forager (Fig. 8), nurse bee (Fig. S9), and queen brains (Fig. S10). Stronger signals were detected with the ‘exon probe’ than with the ‘intron probe’, consistent with the predicted the properties of the *AmMESK2* gene structure. Similar *AmMESK2*-expression pattern was also observed in the brains of drones, which have larger compound eyes and OLs than those of workers or queens (Fig. 9). In the drone brain, while weak signals were detected in most of the brain cortex except the MBs (Fig. 9B), the strongest signals were detected in some dozens of cells in the ventral part of the cortex between the lamina and medulla (Fig. 9E). No significant signals were detected in the other brain regions, including the MB (Fig. 9B, F), cells located surrounded by the OL, MB and SOG (Fig. 9B, G), and cells around the antennal lobes (Fig. 9B, H). These signals were not detected in sections hybridized with a sense probe (Fig. 9C), indicating that the signals represented *AmMESK2*-expression. Weaker signals were also detected in the dorsal part of the OLs, suggesting that *AmMESK2* is expressed preferentially, but not specifically, in the ventral region between the lamina and medulla of the OLs in the drone brain. Therefore, although *AmMESK2* is weakly expressed in most of the brain cortex, it is expressed preferentially in restricted neural cell types, suggesting the importance of this gene product in these cells (Fig. 9B, C).

*AmMESK2* was expressed differentially between the ventral and dorsal parts of the OLs, therefore we also examined whether *AmMESK2* is expressed differentially between the anterior and proximal parts of the OLs by *in situ* hybridization with the horizontal drone brain sections. The *AmMESK2*-expressing cells were located ubiquitously along the anterior-posterior axis and around the outer chasm (Fig. 9I–L). The signals were not detected between the medulla and lobula, confirming that *AmMESK2* was expressed preferentially in neurons located between the lamina and medulla in the honeybee brain.

Finally, we performed real-time RT-PCR to compare the amount of *Amfutsch-, Amtau-* and *AmMESK2* transcripts between the brains of nurse bees and foragers, in which the MB structure changes depending on the division of labor of the workers and correlates with the foraging experience of the foragers [28], [29]. The amount of *Amfutsch-* and *Amtau*-transcripts did not differ significantly between the brains of nurse bees and foragers, whereas the amount of *AmMESK2*-transcript was 1.5-fold higher in the forager brain then in the nurse bee brain (Supporting Information S1, Fig. S11).

## Discussion

The present study is the first to identify genes whose expression was more enriched in the OLs than in the other regions in the honeybee brain. The findings indicated three genes with neural cell type-preferential gene expression profiles in the OLs. To our knowledge, this is the first identification of genes that are expressed in a monopolar cell- (*Amfutsch* and *Amtau*) or ventral medulla-preferential manner (*AmMESK2*) in insect OLs.

One of these genes was a homologue of a gene for microtubule-associated protein, *futsch*/*map1*. Both mammalian MAP1 and *Drosophila* Futsch function to stabilize axon structures by binding to microtubules at axons or axon terminals [45], [50]. Although the MAP1 family contains several genes in mammals, in *Drosophila*, MAP1 function appears to be fulfilled by a single gene, *futsch* [44]. Similarly, *Amfutsch* was a single copy gene in the honeybee genome. In *Drosophila*, *Dmfutsch* encodes the 22C10 antigen, which has been widely used as a neuronal marker [51]. In adult brain, 22C10-immunoreactivity is detected in some central nervous systems, such as chiasmas or antennal nerves, as well as in most peripheral neurons [52], whereas *Amfutsch* was expressed preferentially in the monopolar cells in the lamina of the adult honeybee brain. In addition, double *in situ* hybridization showed that *futsch* and *tau*, which functionally overlap in mice and flies, are co-expressed in the monopolar cells in the lamina. Similar to the situation for *Amfutsch*, although the Tau family contains several genes in mammals, *tau* represents a single copy gene in both *Drosophila* and the honeybee. Furthermore, although *Dmtau* is expressed in photoreceptors in *Drosophila* adults and as well as in brain and most peripheral neurons in *Drosophila* larvae [46], *Amtau* was expressed preferentially in the monopolar cells in the lamina of the adult honeybee brain. These results suggested that *Amfutsch* and *Amtau* are involved in the monopolar cell-specific cell characteristics or structures. Furthermore, the expression profiles of *Dmfutsch* and *Dmtau* in the adult brain seem distinct from those of *Amfutsch* and *Amtau*, suggesting that both *futsch-* and *tau*-expression might be differently regulated among these insect species. Considering that monopolar cells may be involved in detection of contrast of visual objects, and project axons into the medulla as well as into the lobula, *Am*Futsch and *Am*Tau might play important roles in stabilizing axon structures, which is essential for neural functions of monopolar cells.

*In situ* hybridization of *Amfutsch* and *Amtau* using developing pupal brain sections indicated that, although both genes were expressed in the pupal brains, they were differentially expressed in the pupal brains, suggesting that these genes function in a brain-region dependent manner during pupal stages (Supporting Information S1, Fig. S5 and S6). Considering that expression of both *Amfutsch* and *Amtau* is enriched in monopolar cells, it might be that the axons of monopolar cells are solid or undergo remodeling under certain circumstances, which requires more abundant *Am*Futsch and *Am*Tau than the other brain regions. Because essentially the same *Amfutsch*- and *Amtau*-expression profiles were detected in all forager, nurse bee, queen, and drone brains, it is likely that *Amfutsch* and *Amtau* function similarly in monopolar cells irrespective of the sex, caste, or division of labor of workers.

The function of *Amfutsch* and *Amtau* in the cells with large somata that are located beneath the MB calyces remains unknown. Based on the location of these cells, they might be neurosecretory cells. Axonal stabilization might also be required in these cell types. In the honeybee, immunoreactivity for pigment dispersing hormone, which is involved in insect circadian clock, is detected in neurons located at the medial margin of medulla as well as in large neurons located beneath the MBs [53]. Although the latter cells resemble to those expressing *Amfutsch* and *Amtau*, the former cells differ from the monopolar cells, Thus, the relationship between the circadian clock and the *Amfutsch*- and *Amtau*-expressing cells is unclear, at present. Finally, *Amtau* was not identified in our cDNA microarray screening as a gene whose expression was more enriched in the OLs than in the other brain regions, possibly because our cDNA microarray contained only 5000 cDNA subclones corresponding to the differential display-positive bands, and thus the variety of genes printed on the cDNA microarray was restricted.

The second gene was a homologue of *MESK2*. *DmMESK2* was originally identified while screening for genes that have potentials to modulate RAS-signaling [44]: this gene enhances KSR-signaling, which functions downstream of RAS1, when misexpressed in the *Drosophila* retina. Its actual function and expression profile in the adult brain has not been analyzed in *Drosophila*. Because there was only a single gene for *MESK2* in both honeybee and *Drosophila*, we concluded that *AmMESK2* is a homologue of *DmMESK2*. The conserved Ndr domain of *Am*MESK2 suggests that *Am*MESK2 has molecular functions similar to those of *Dm*MESK2. Interestingly, *AmMESK2* was expressed strongly in only a few dozen cells located in the ventral region between the lamina and medulla in the OLs. Analysis of *AmMESK2* expression revealed that the *AmMESK2*-expressing cells form a zone at the ventral part that spans from the anterior to the posterior regions of the OLs, close to the outer chiasm. It might be that these cells are important for detecting visual cues, e.g., optic flow, present on the ground rather than in the air. Although the function of neurons expressing *AmMESK2* is currently unknown, *AmMESK2* may be involved in some kind of neural plasticity by modulating RAS-signaling. These findings indicate that the medulla contains a new subclass of cells characterized by *AmMESK2*-expression, which has not been identified by previous anatomic or electrophysiologic studies. Neurons in the lamina and medulla form ‘cartridges’ and ‘columns’, each of which comprise several types of neurons [7], [20]–[23]. Thus, it is possible that the *AmMESK2*-expressing cells comprise a part of the ‘cartridges’ or ‘columns’, and that these ‘cartridges’ or ‘columns’ represent a considerable part of the ventral region between the lamina and medulla in the OLs.

Whether monopolar cell-preferential *Amfutsch/Amtau*-expression and ventral medulla-preferential *AmMESK2*-expression are restricted to honeybees, are conserved among a part of social Hymenopteran insects, or are conserved among various Hymenopteran insects, requires further investigation. From the view of ‘molecular dissection’ of the honeybee brain, *Amfutsch*/*Amtau* and *AmMESK2* may be useful tools for detecting axons of the cells that express these genes: these genes aid in visualizing axons of monopolar cells and ventral medulla cells that express *AmMESK2*, for example, by immunochemical staining or by using reporter genes in future experiments. Considering that the MB structure changes depending on the division of labor of the workers and correlates with the foraging experience of the foragers [51], [52], it is plausible that visual experience affects patterns or densities of axonal projections of OL neurons to the MBs. In fact, the amount of *AmMESK2*-transcript was 1.5-fold higher in the forager brains than in the nurse bee brains, raising the possibility that the function of *Am*MEKS2 is more necessary in the forager brain than in the nurse bee brain (Supporting Information S1, Fig. S11). Further studies to examine whether projection patterns of the monopolar cells and ventral medulla neurons expressing *Amfutsch*/*Amtau* and *AmMESK2* depend on the foraging flight of workers can be performed using *Amfutsch*/*Amtau* and *AmMESK2* as cell type-preferential markers.

## Materials and Methods

### Animals

European honeybees (*Apis mellifera* L.) were purchased from a local dealer and maintained at the University of Tokyo. Foragers with pollen loads were captured at the hive entrance. Nurse bees were collected when they were feeding their brood in honeycombs [26], [54]. Drones were collected in the hive. Queens were purchased from the same local dealer.

### cDNA Microarray Analysis

A cDNA microarray was performed as described previously [39] with some modifications. We previously prepared a cDNA microarray with over 5000 cDNA subclones representing various genes expressed in the adult honeybee brains: the subclones were cloned from gel portions corresponding to the positive bands in the differential display method used to identify genes expressed in honeybee brain in a brain region- or role-preferential manner [35]. In the present study, we used this cDNA microarray to compare gene expression profiles between the OLs and the other brain regions. Total RNA was extracted from the OLs and the remaining brain regions dissected from the heads of 79 foragers using TRIzol (Invitrogen). Total RNA (500 ng) from the OLs and the remaining brain regions were amplified using an Amino Allyl MessageAmp aRNA Amplification kit (Ambion). Total RNA from the OLs and the other brain regions was divided into 4 groups and two groups were labeled with fluorescent dye Cy5, while the other two groups were labeled with Cy3 (Amersham Bioscience), to prepare two sets of Cy5- or Cy3-labeled RNA from the OLs and two sets of Cy5- or Cy3-labeled RNA from the other brain regions. Hybridization was performed twice using a pair of ‘Cy5-labeled OL RNA and Cy3-labeled the other brain region RNA’, and a pair of ‘Cy3-labeled OL RNA and Cy5-labeled the other brain region RNA’. Data analyses were performed using Genespring software (Silicon Genetics). Hybridization was performed twice by exchanging the dyes, Cy5 or Cy3, that were used to label the RNAs, and this hybridization process was repeated to confirm the results.

We calculated the ratio of the expression level of each clone in the OLs relative to that in the remaining brain regions and looked for clones whose ratios were greater than 1.4-fold. Sequencing the positive clones revealed many redundant clones, and 45 independent clones were identified as candidate genes whose expression was more enriched in the OLs than in the other brain regions. An expression analysis of 19 clones selected arbitrarily from the 45 identified candidate clones performed using *in situ* hybridization with a DIG-labeled RNA probe led to the identification of two clones, Clones #1 and #2 (GenBank accession Nos. BP538943 and BP539264), that were strongly expressed in the OLs compared with the other brain regions. Expression of 15 of the remaining 17 candidates was not clearly detected in any brain region by *in situ* hybridization, possibly because their expression levels were below the detection threshold, whereas the expression of the remaining 2 candidates was detected in both the OLs and other brain regions in the honeybee brain, suggesting that these genes represented ‘false positive clones’.

### In Situ Hybridization Analysis

*In situ* hybridization was performed as described previously with some modifications [34], [54], [55]. Frozen vertical brain sections (10 µm thick) were fixed in 4% paraformaldehyde in phosphate buffer, pretreated, and hybridized with digoxigenin (DIG)-labeled riboprobes. The DIG-labeled riboprobes were synthesized by T7 or SP6 polymerase with a DIG labeling mix (Roche) from template cDNAs containing the fragment cDNA for Clone #1 (BP538943, which corresponds to +9883 to +10283 of *GB11509/Amfutsch*), *Amtau* (+2689 to +3103 of *hmm14986*), Clone #2 (BP539264, which corresponds to +213115 to +212462 of LG6/putative intron of *AmMESK2*), and ‘exon probe’ of *AmMESK2* (+44 to +1052 of *GB18470*), respectively. After stringent washes, DIG-labeled riboprobes were detected immunocytochemically with alkaline phosphatase-conjugated anti-DIG antibody using a DIG Nucleic Acid Detection Kit (Roche). To examine the monopolar cell-preferential expression or co-expression of *Amfutsch* with *Amtau* in monopolar cells, the *Amfutsch* riboprobe was labeled with DIG and the *Amtau* riboprobe was labeled with biotin. DIG-labeled *Amfutsch* riboprobes were detected with the HNPP Fluorescent Detection Set (Roche), and biotin-labeled *Amtau* riboprobes were detected with the TSA plus System (Perkin Elmer). As a negative control, sections were hybridized with sense probes and the antisense probe-specific signals were confirmed in every experiment. Micrographs of fluorescent *in situ* hybridization were taken using a fluorescent microscope (Axio Imager Z1, Carl Zeiss). 4′,6-Diamino-2-phenylindole, dihydrochloride (DAPI, Invitrogen) was used to stain the nuclear DNA [55], [56]. Intensity and brightness of the micrographs were processed with Photoshop software (Adobe Systems).

## Supporting Information

Supporting Information S1 (0.06 MB DOC)

Figure S1 *In situ* hybridization of *Amfutsch* in the forager brains. *In situ* hybridization using DIG-labeled RNA antisense (B, D–I) and sense (C) *Amfutsch* probes with forager brain sections. (A) Schematic representation of the signals detected in the left-brain hemisphere of the forager brain. Black circles indicate the stronger signals. (D–I) Magnified views of parts of (B) corresponding to the boxes shown in (A). The stronger signals detected in the lamina (D, E) and in another region (H) are indicated by black arrowheads. White arrowheads indicate regions with no signals (E–I). Black arrows indicated intermediate signals near the SOG (I). Scale bars = 100 Âµm. Asterisks indicate non-specific staining. D, dorsal; L, lateral; la, lamina; lCa, lateral calyx; lo, lobula; M, medial; me, medulla; mCa, medial calyx; Re, retina; SOG, subesophageal ganglion; V, ventral.
(5.45 MB TIF)

Figure S2 *In situ* hybridization of *Amfutsch* in the queen brains. *In situ* hybridization using DIG-labeled RNA antisense (B, D–I) and sense (C) *Amfutsch* probes and the queen brain sections. (A) Schematic representation of the signals detected in the left-brain hemisphere of the queen brain. Black circles and black check marks indicate the stronger and intermediate signals, respectively. (D–I) Magnified views of parts of (B) corresponding to the boxes shown in (A). The stronger signals detected in the lamina (D, E) and in another region (H) are indicated by black arrowheads. White arrowheads indicated the regions with no signals (D–I). Black arrows indicate intermediate signals near the MBs (G) and the SOG (I). Scale bars = 100 Âµm. Asterisks indicate non-specific staining. D, dorsal; L, lateral; la, lamina; lCa, lateral calyx; lo, lobula; M, medial; me, medulla; mCa, medial calyx; Re, retina; SOG, subesophageal ganglion; V, ventral.
(5.49 MB TIF)

Figure S3 *In situ* hybridization of *Amfutsch* in the drone brains. *In situ* hybridization using DIG-labeled RNA antisense (B, D–I) and sense (C) *Amfutsch* probes with drone brain sections. (A) Schematic representation of the signals detected in the left-brain hemisphere of the drone brain. Black circles and black check marks indicate the stronger and intermediate signals, respectively. (D–G) Magnified views of parts of (B) corresponding to the boxes shown in (A). The stronger signals detected in the lamina (D, E) and in another region (F) are indicated by black arrowheads. White arrowheads indicate the regions with no signals (D–F). Black arrows indicate intermediate signals in regions near the MBs (F) and SOG (G). Scale bars = 100 Âµm. Asterisks indicate non-specific staining. AL, antennal lobe; D, dorsal; L, lateral; la, lamina; lCa, lateral calyx; lo, lobula; M, medial; me, medulla; mCa, medial calyx; Re, retina; V, ventral.
(4.55 MB TIF)

Figure S4 *In situ* hybridization of *Amtau* in the nurse bee brains. *In situ* hybridization using DIG-labeled RNA antisense (B, D–I) and sense (C) *Amtau* probes with nurse bee brain sections. (A) Schematic representation of signals detected in the left-brain hemisphere of the forager brain. Black circles indicate stronger signals. (D–I) Magnified views of parts of (B) corresponding to the boxes shown in (A). (J) Magnified view of the same part as (I) of another section, which includes intermediate signals. The stronger signals detected in the lamina (D, E) and the other region (H) are indicated by black arrowheads. White arrowheads indicate the regions with no signals (E–J). Black arrows indicated intermediate signals near the SOG (J). Scale bars = 100 Âµm. Asterisks indicate non-specific staining. D, dorsal; L, lateral; la, lamina; lCa, lateral calyx; lo, lobula; M, medial; me, medulla; mCa, medial calyx; Re, retina; SOG, subesophageal ganglion; V, ventral.
(5.54 MB TIF)

Figure S5 Expression analysis of *Amfutsch* in the developing pupal brain. *In situ* hybridization using DIG-labeled RNA *Amfutsch* antisense probes with developing pupal brain sections (Stage P2, P4, and P5). (A) Schematic representation of signals detected in the left hemisphere of the developing pupal brain. Grey regions indicate the part of the brain cortex with stronger signals. (B–D) Results of in situ hybridization using developing pupal brain sections at the P2, P4, and P5 stages [S1], respectively (for staging, also see legend for Fig. S6). Note that relatively strong signals were detected in almost the whole brain cortex, whereas only weak signals were detected in the developing MB regions surrounded by dotted lines [S1, 3, 4]. We could not identify the monopolar cells undergoing differentiation in these developing pupal brain sections. Scale bars = 100 Âµm. D: dorsal, L: lateral, M: medial, MB: mushroom body, OL: optic lobe, V: ventral.
(3.75 MB TIF)

Figure S6 Expression analysis of *Amtau* in the developing pupal brain. *In situ* hybridization using DIG-labeled RNA antisense *Amtau* probes with developing worker brain sections. (A) Results of the *in situ* hybridization using a section from the right hemisphere of the developing pupal brain. (B) A magnified view of the right pupal MB, indicated by the box in panel (A). (C, D) Schematic representation of signals detected in the right hemisphere of the developing pupal brain, which correspond to panels (A) and (B), respectively. Black circles indicate stronger signals. Gray regions indicate brain cortex with medium signals. Proliferating MB cells are represented by open circles in the inner core of the inside of developing calyces, and are indicated by arrows. (E upper panel) Time-course of the developmental stages, including the larva, prepupa, pupa (P1–9), and adult. (E lower panels) Magnified views of the in situ hybridization of the developing pupal MBs at stages P1, P2, P4, and P5 [S1]. Stronger signals were detected around the proliferative MB cells, indicated by arrows. Scale bars = 100 µm. D: dorsal, L: lateral, M: medial, MB: mushroom body, OL: optic lobe, Pe: peduncle, V: ventral.
(3.75 MB TIF)

Figure S7 Amplification of the cDNA fragment that contained both Clone #2 and the predicted exon region of *AmMESK2*. (A) The predicted gene structure of *AmMESK2* (GB18470, middle line) is indicated below the Linkage group 6.13 (upperline), where the putative exons of *AmMESK2* are indicated with vertical solid boxes. Numbers above the Linkage group indicate nucleotide positions. Positions of Clone #2 and primers used to amplify the cDNA fragment that contained both Clone #2 and the predicted 6th exon region of *AmMESK2* are indicated with arrowheads and solid box, respectively. Their structure of the ‘exon probe’ is indicated below the *AmMESK2* gene structure (lower panel). (B) Agarose gel electrophoresis of the cDNA fragment that contained both Clone #2 and the putative 6th exon of *AmMESK2*, amplified by RT-PCR using the honeybee total brain RNA and the primer set described in panel (A). The detected band position (approximately 700 bp), which coincides with the predicted size (678 bp), is indicated by an arrowhead at the left of the panel. The numbers at the right of the panel indicate sizes of the molecular mass makers in bp. Note that a band of the predicted size was detected in the RT+ lane, but not in the RT- lane.
(1.30 MB TIF)

Figure S8 *In situ* hybridization with the intron probe of *AmMESK2* in the nurse bee brains. *In situ* hybridization using DIG-labeled RNA antisense (B, D–H) and sense (C) *AmMESK2* probes with nurse bee brain sections. (A) Schematic representation of signals detected in the left-brain hemisphere of the nurse bee brain. Black circles indicate stronger signals. (D–H) Magnified views of pars of (B) corresponding to the boxes shown in (A). The signals detected in the cortex between the lamina and medulla are indicated by black arrowheads. Scale bars = 100 µm. D, dorsal; L, lateral; AL, antennal lobe; lCa, lateral calyx; M, medial; me, medulla; mCa, medial calyx; Re, retina; V, ventral.
(4.23 MB TIF)

Figure S9 *In situ* hybridization with the exon probe of *AmMESK2* in the nurse bee brains. *In situ* hybridization using DIG-labeled RNA antisense (B, D–H) and sense (C) *AmMESK2* probes with nurse bee brain sections. (A) Schematic representation of signals detected in the left-brain hemisphere of the nurse bee brain. Black circles indicate stronger signals. (D–H) Magnified views of parts of (B) corresponding to the boxes shown in (A). The stronger signals detected between the lamina and medulla are indicated by black arrowheads. Scale bars = 100 µm. Asterisks indicate non-specific staining. AL, antennal lobe; D, dorsal; L, lateral; la, lamina; lCa, lateral calyx; M, medial; me, medulla; mCa, medial calyx; OL, optic lobe; Re, retina; V, ventral.
(4.52 MB TIF)

Figure S10 *In situ* hybridization with the exon probe of *AmMESK2* in the queen brains. *In situ* hybridization using DIG-labeled RNA antisense (B, D–H) and sense (C) *AmMESK2* probes with queen brain sections. (A) Schematic representation of signals detected in the left-brain hemisphere of the queen brain. Black circles indicate stronger signals. (D–H) Magnified views of parts of (B) corresponding to the boxes shown in (A). The stronger signals detected between the lamina and medulla are indicated by black arrowheads. Scale bars = 100 µm. Asterisks indicate non-specific staining. AL, antennal lobe; D, dorsal; L, lateral; la, lamina; lCa, lateral calyx; M, medial; me, medulla; mCa, medial calyx; OL, optic lobe; Re, retina; V, ventral.
(4.14 MB TIF)

Figure S11 Comparison of the amounts of *Amfutsch*-, *Amtau*-, and *AmMESK2*-transcripts in the whole brains of queens, nurse bees, and foragers. Real-time RT-PCR was performed to compare the amounts of *Amfutsch*-, *Amtau*-, and *AmMESK2*-transcripts in the whole brains of nurse bees and foragers. N: nurse bee, F: forager. The amount of *Amfutsch*-, *Amtau*-, and *AmMESK2*-transcripts were normalized with that of *actin* defining the average of normalized mRNA levels in the nurse bee as 1. An asterisk indicates a significant difference between nurse bees and foragers (P<0.05; Welch's t-test).
(1.90 MB TIF)
