# Supplementary material for: In Situ Hybridization Analysis of the Expression of Futsch, Tau, and MESK2 Homologues in the Brain of the European Honeybee (Apis mellifera L.)
Source: PLoS One. 2010 Feb 16;5(2):e9213. doi: 10.1371/journal.pone.0009213 (PMC2821913; doi:10.1371/journal.pone.0009213)
Supplement: Supporting Information S1 — (0.06 MB DOC) [file pone.0009213.s001.doc]

**Supporting Information S1**

**Materials and Methods for Figure S5-7, and S11**

*In situ hybridization analysis of Amfutsch and Amtau in the developing pupal brain*

*In situ* hybridization of *Amfutsch* and *Amtau* was performed as described in the Materials and Methods of the main text, using DIG-labeled riboprobes and brain sections of worker pupae at stages P2, P4, and P5 (for *Amfutsch*) and P1, P2, P4, and P5 (for *Amtau*). Pupal stages were determined according to Ganeshina et al. [S1].

*Amplification of the cDNA fragment that contained both Clone #2 and the predicted exon region of AmMESK2*

Total brain RNA was extracted from whole worker brains using TRIzol (Invitrogen), as described previously [S2]. After reverse transcription of the equal amounts of total RNA with (RT+) or without (RT-) Superscript II (Invitrogen), the cDNA was amplified by PCR using the following primers: antisense primer, +74 to +93 of Clone #2; sense primer, +982 to +1001 of *GB18470,* corresponding to the predicted 6th exon of *AmMESK2*. The resulting PCR product was subjected to agarose gel electrophoresis, followed by subcloning into a pGEM T-easy vector and sequencing.

*Comparison of the amounts of Amfutsch-, Amtau- and AmMESK2-transcripts in the whole brains of nurse bees and foragers*

Real-time RT-PCR was performed to compare the amounts of *Amfutsch-*, *Amtau-,* and *AmMESK2*-transcripts in the whole brains of nurse bees and foragers. Total RNA was extracted from the whole brains of nurse bees (3 lots, each lot included 5 individuals) and foragers (3 lots, each lot included 5 individuals) using TRIzol, and subjected to real-time RT-PCR using SYBR Premix Ex Taq II (Perfect Real Time; TaKaRa), essentially as described previously [S2]. The gene-specific primer sets used were: +1480 to +1500 and +1572 to +1592 of *Amfutsch*; +2689 to +2708 and +2765 to +2786 of *hmm14986* for *Amtau*; +416 to +436 and +493 to +512 of *GB18470* for *AmMESK2* and 5’-GCCGTGATTTGACTGACTAC-3’ and 5’-GATGCAGCAGTTGCCATTTC-3’ for *actin*.

**References for Figures S5-7 and S11**

S1. Ganeshina O, Schäfer S, Malun D (2000) Proliferation and programmed cell death of neuronal precursors in the mushroom bodies of the honeybee. J Comp Neurol 417: 349-365.

S2. Ueno T, Nakaoka T, Takeuchi H, Kubo T (2009) Differential gene expressions in the hypopharyngeal glands of worker honeybees (*Apis mellifera* L.) associated with the age-dependent role change. Zool Sci 26: 557-563.

S3. Farris SM, Sinakevitch I (2003) Development and evolution of the insect mushroom bodies: towards the understanding of conserved developmental mechanisms in a higher brain center. Arthropod Structure and Dev 32: 79-101.

S4. Farris SM, Abrams AI, Strausfeld NJ (2004) Development and morphology of class II Kenyon cells in the mushroom bodies of the honeybee, *Apis mellifera*. J Comp Neurol 474: 325-339.
