# Supplementary figures and images for: In Situ Hybridization Analysis of the Expression of Futsch, Tau, and MESK2 Homologues in the Brain of the European Honeybee (Apis mellifera L.)
Source: PLoS One. 2010 Feb 16;5(2):e9213. doi: 10.1371/journal.pone.0009213 (PMC2821913; doi:10.1371/journal.pone.0009213)

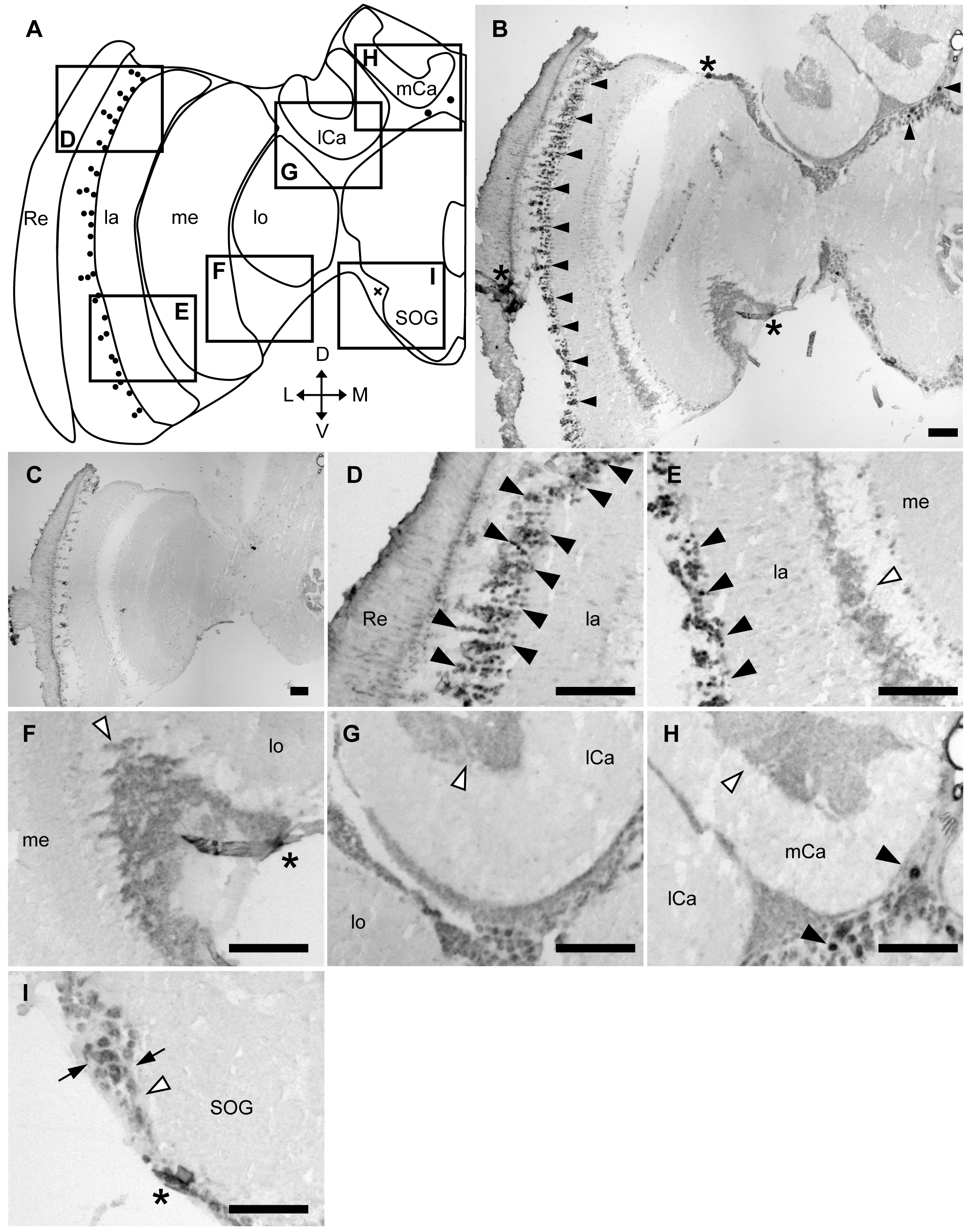

Supplement: Figure S1 — In situ hybridization of Amfutsch in the forager brains. In situ hybridization using DIG-labeled RNA antisense (B, D–I) and sense (C) Amfutsch probes with forager brain sections. (A) Schematic representation of the signals detected in the left-brain hemisphere of the forager brain. Black circles indicate the stronger signals. (D–I) Magnified views of parts of (B) corresponding to the boxes shown in (A). The stronger signals detected in the lamina (D, E) and in another region (H) are indicated by black arrowheads. White arrowheads indicate regions with no signals (E–I). Black arrows indicated intermediate signals near the SOG (I). Scale bars = 100 Âµm. Asterisks indicate non-specific staining. D, dorsal; L, lateral; la, lamina; lCa, lateral calyx; lo, lobula; M, medial; me, medulla; mCa, medial calyx; Re, retina; SOG, subesophageal ganglion; V, ventral. (5.45 MB TIF) [file pone.0009213.s002.tif]

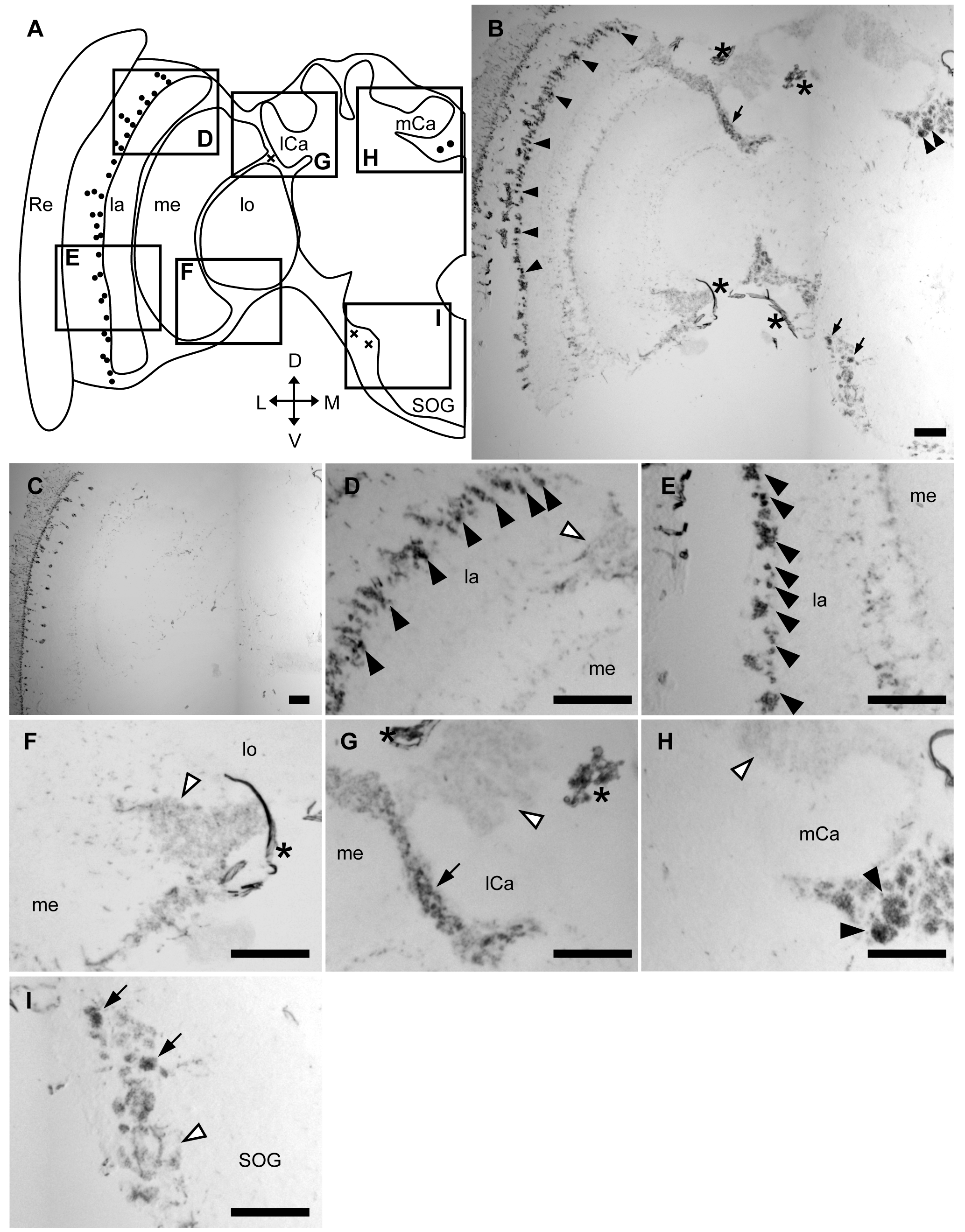

Supplement: Figure S2 — In situ hybridization of Amfutsch in the queen brains. In situ hybridization using DIG-labeled RNA antisense (B, D–I) and sense (C) Amfutsch probes and the queen brain sections. (A) Schematic representation of the signals detected in the left-brain hemisphere of the queen brain. Black circles and black check marks indicate the stronger and intermediate signals, respectively. (D–I) Magnified views of parts of (B) corresponding to the boxes shown in (A). The stronger signals detected in the lamina (D, E) and in another region (H) are indicated by black arrowheads. White arrowheads indicated the regions with no signals (D–I). Black arrows indicate intermediate signals near the MBs (G) and the SOG (I). Scale bars = 100 Âµm. Asterisks indicate non-specific staining. D, dorsal; L, lateral; la, lamina; lCa, lateral calyx; lo, lobula; M, medial; me, medulla; mCa, medial calyx; Re, retina; SOG, subesophageal ganglion; V, ventral. (5.49 MB TIF) [file pone.0009213.s003.tif]

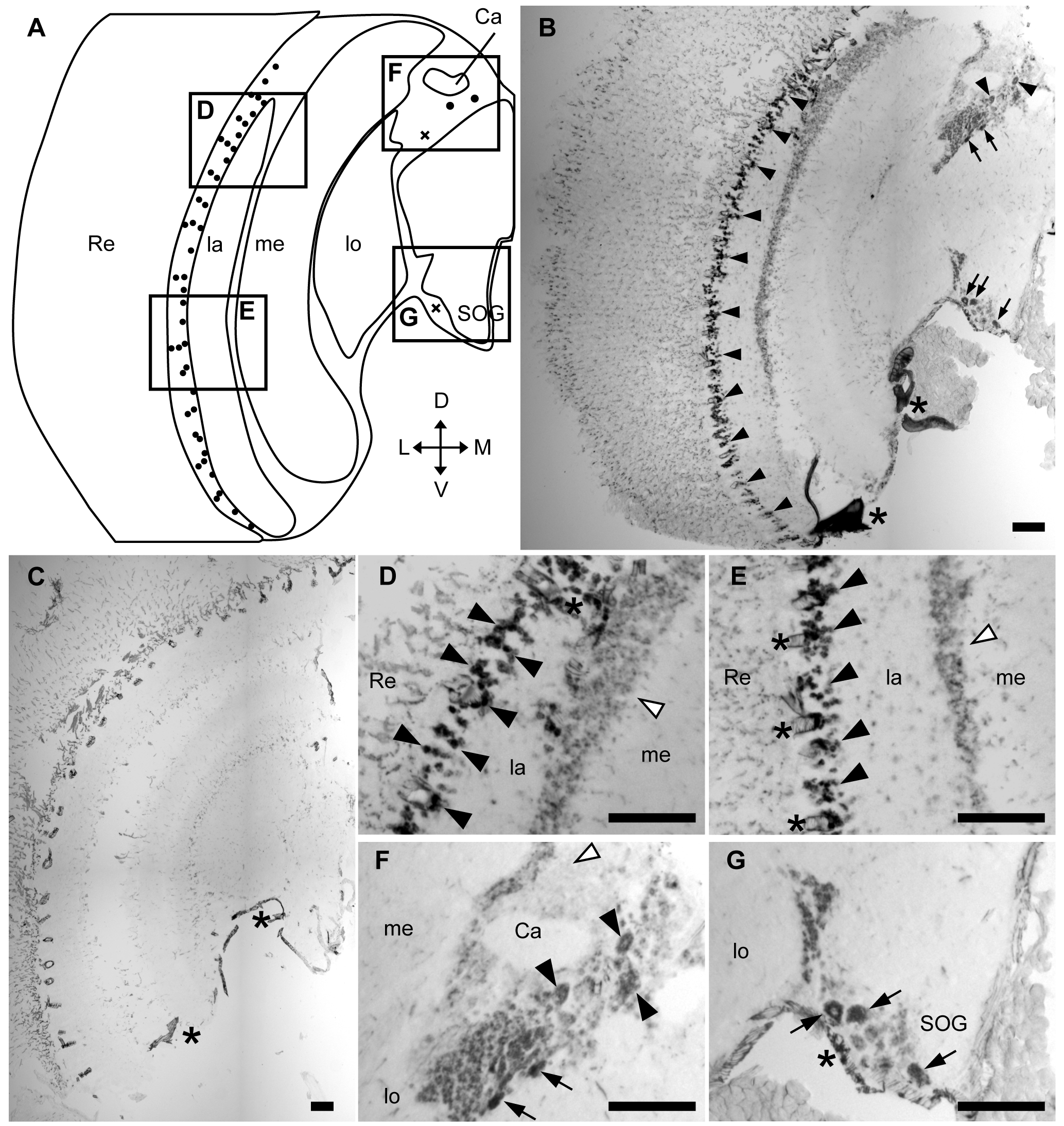

Supplement: Figure S3 — In situ hybridization of Amfutsch in the drone brains. In situ hybridization using DIG-labeled RNA antisense (B, D–I) and sense (C) Amfutsch probes with drone brain sections. (A) Schematic representation of the signals detected in the left-brain hemisphere of the drone brain. Black circles and black check marks indicate the stronger and intermediate signals, respectively. (D–G) Magnified views of parts of (B) corresponding to the boxes shown in (A). The stronger signals detected in the lamina (D, E) and in another region (F) are indicated by black arrowheads. White arrowheads indicate the regions with no signals (D–F). Black arrows indicate intermediate signals in regions near the MBs (F) and SOG (G). Scale bars = 100 Âµm. Asterisks indicate non-specific staining. AL, antennal lobe; D, dorsal; L, lateral; la, lamina; lCa, lateral calyx; lo, lobula; M, medial; me, medulla; mCa, medial calyx; Re, retina; V, ventral. (4.55 MB TIF) [file pone.0009213.s004.tif]

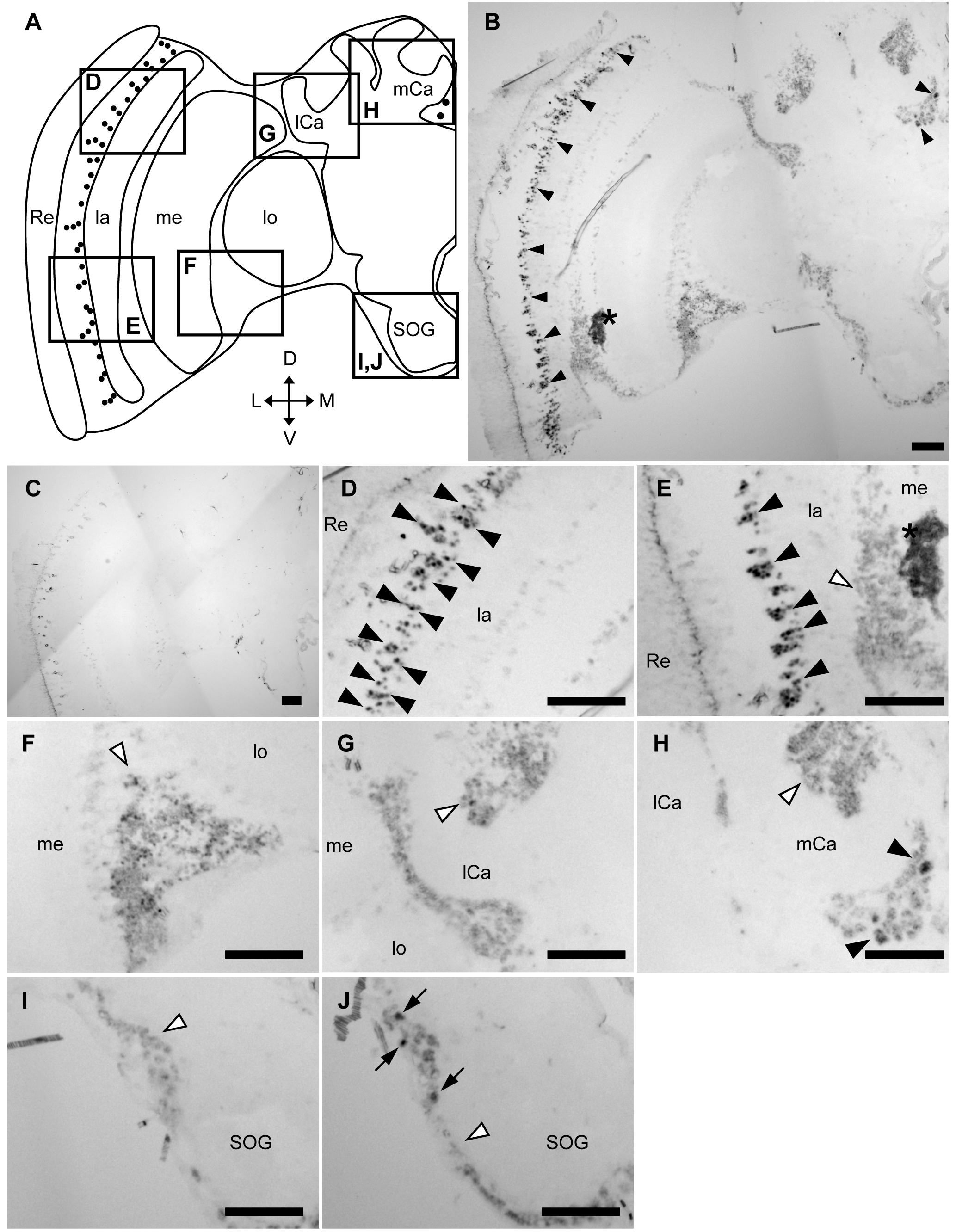

Supplement: Figure S4 — In situ hybridization of Amtau in the nurse bee brains. In situ hybridization using DIG-labeled RNA antisense (B, D–I) and sense (C) Amtau probes with nurse bee brain sections. (A) Schematic representation of signals detected in the left-brain hemisphere of the forager brain. Black circles indicate stronger signals. (D–I) Magnified views of parts of (B) corresponding to the boxes shown in (A). (J) Magnified view of the same part as (I) of another section, which includes intermediate signals. The stronger signals detected in the lamina (D, E) and the other region (H) are indicated by black arrowheads. White arrowheads indicate the regions with no signals (E–J). Black arrows indicated intermediate signals near the SOG (J). Scale bars = 100 Âµm. Asterisks indicate non-specific staining. D, dorsal; L, lateral; la, lamina; lCa, lateral calyx; lo, lobula; M, medial; me, medulla; mCa, medial calyx; Re, retina; SOG, subesophageal ganglion; V, ventral. (5.54 MB TIF) [file pone.0009213.s005.tif]

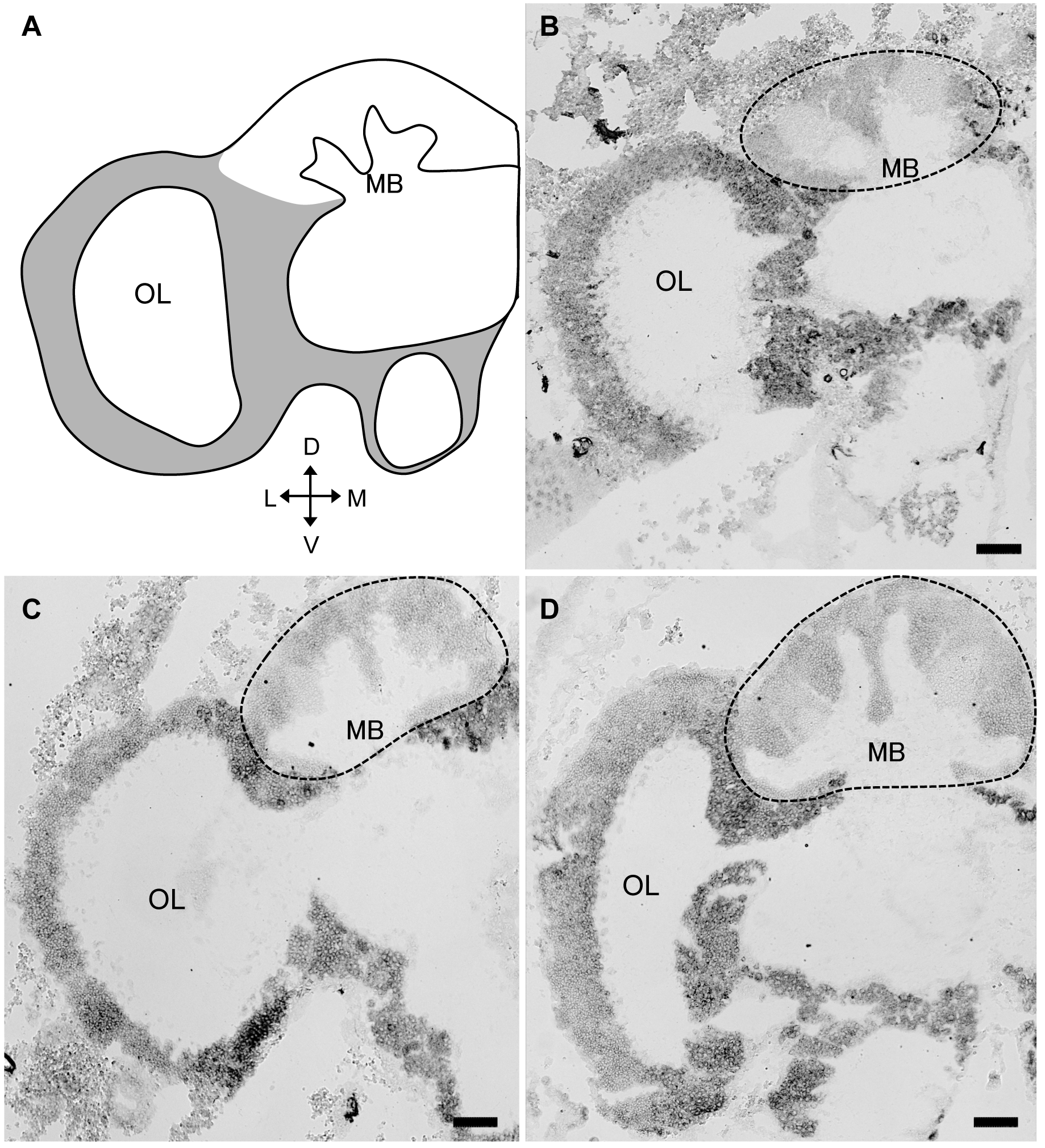

Supplement: Figure S5 — Expression analysis of Amfutsch in the developing pupal brain. In situ hybridization using DIG-labeled RNA Amfutsch antisense probes with developing pupal brain sections (Stage P2, P4, and P5). (A) Schematic representation of signals detected in the left hemisphere of the developing pupal brain. Grey regions indicate the part of the brain cortex with stronger signals. (B–D) Results of in situ hybridization using developing pupal brain sections at the P2, P4, and P5 stages [S1], respectively (for staging, also see legend for Fig. S6). Note that relatively strong signals were detected in almost the whole brain cortex, whereas only weak signals were detected in the developing MB regions surrounded by dotted lines [S1, 3, 4]. We could not identify the monopolar cells undergoing differentiation in these developing pupal brain sections. Scale bars = 100 Âµm. D: dorsal, L: lateral, M: medial, MB: mushroom body, OL: optic lobe, V: ventral. (3.75 MB TIF) [file pone.0009213.s006.tif]

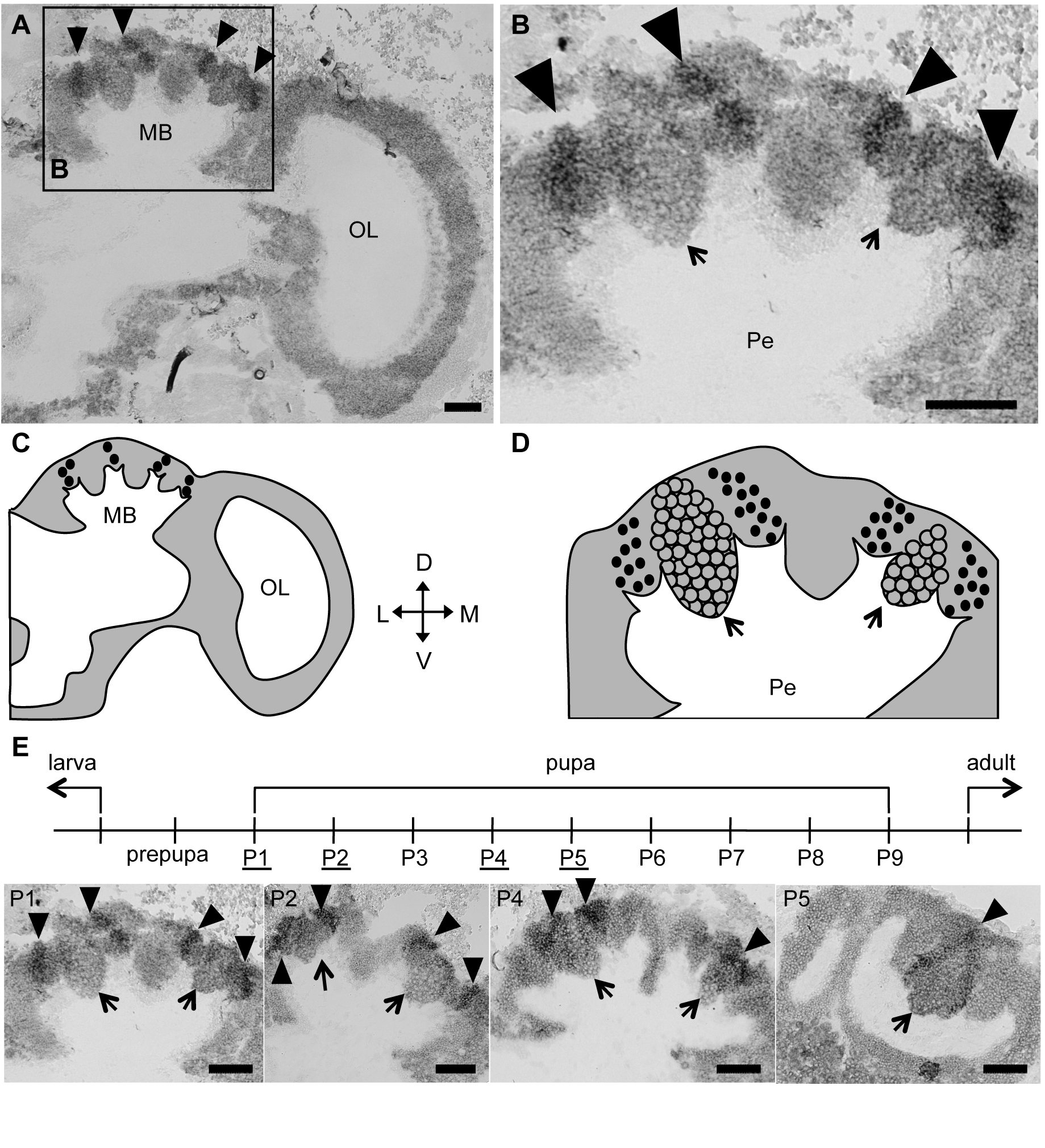

Supplement: Figure S6 — Expression analysis of Amtau in the developing pupal brain. In situ hybridization using DIG-labeled RNA antisense Amtau probes with developing worker brain sections. (A) Results of the in situ hybridization using a section from the right hemisphere of the developing pupal brain. (B) A magnified view of the right pupal MB, indicated by the box in panel (A). (C, D) Schematic representation of signals detected in the right hemisphere of the developing pupal brain, which correspond to panels (A) and (B), respectively. Black circles indicate stronger signals. Gray regions indicate brain cortex with medium signals. Proliferating MB cells are represented by open circles in the inner core of the inside of developing calyces, and are indicated by arrows. (E upper panel) Time-course of the developmental stages, including the larva, prepupa, pupa (P1–9), and adult. (E lower panels) Magnified views of the in situ hybridization of the developing pupal MBs at stages P1, P2, P4, and P5 [S1]. Stronger signals were detected around the proliferative MB cells, indicated by arrows. Scale bars = 100 µm. D: dorsal, L: lateral, M: medial, MB: mushroom body, OL: optic lobe, Pe: peduncle, V: ventral. (3.75 MB TIF) [file pone.0009213.s007.tif]

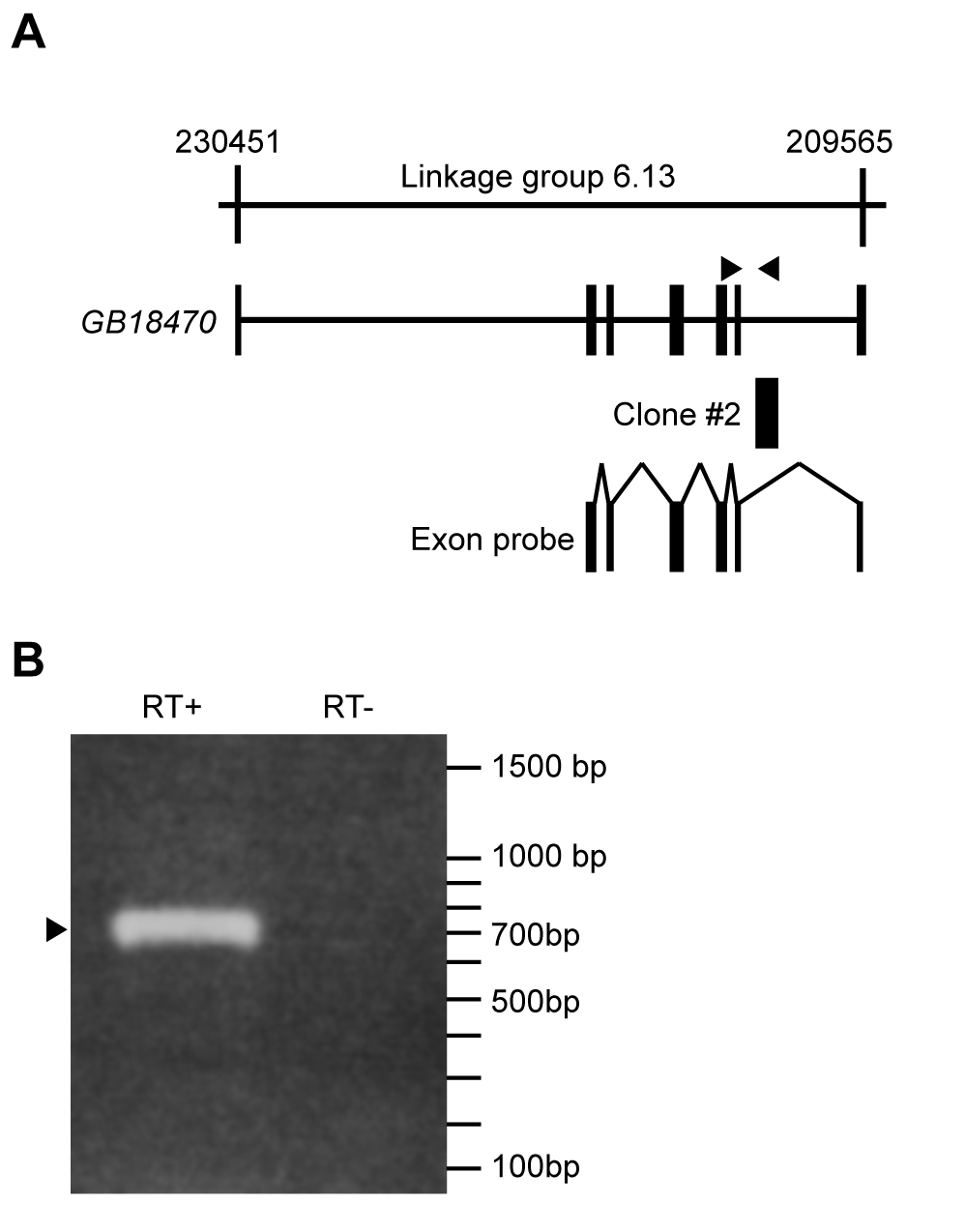

Supplement: Figure S7 — Amplification of the cDNA fragment that contained both Clone #2 and the predicted exon region of AmMESK2. (A) The predicted gene structure of AmMESK2 (GB18470, middle line) is indicated below the Linkage group 6.13 (upperline), where the putative exons of AmMESK2 are indicated with vertical solid boxes. Numbers above the Linkage group indicate nucleotide positions. Positions of Clone #2 and primers used to amplify the cDNA fragment that contained both Clone #2 and the predicted 6th exon region of AmMESK2 are indicated with arrowheads and solid box, respectively. Their structure of the ‘exon probe’ is indicated below the AmMESK2 gene structure (lower panel). (B) Agarose gel electrophoresis of the cDNA fragment that contained both Clone #2 and the putative 6th exon of AmMESK2, amplified by RT-PCR using the honeybee total brain RNA and the primer set described in panel (A). The detected band position (approximately 700 bp), which coincides with the predicted size (678 bp), is indicated by an arrowhead at the left of the panel. The numbers at the right of the panel indicate sizes of the molecular mass makers in bp. Note that a band of the predicted size was detected in the RT+ lane, but not in the RT- lane. (1.30 MB TIF) [file pone.0009213.s008.tif]

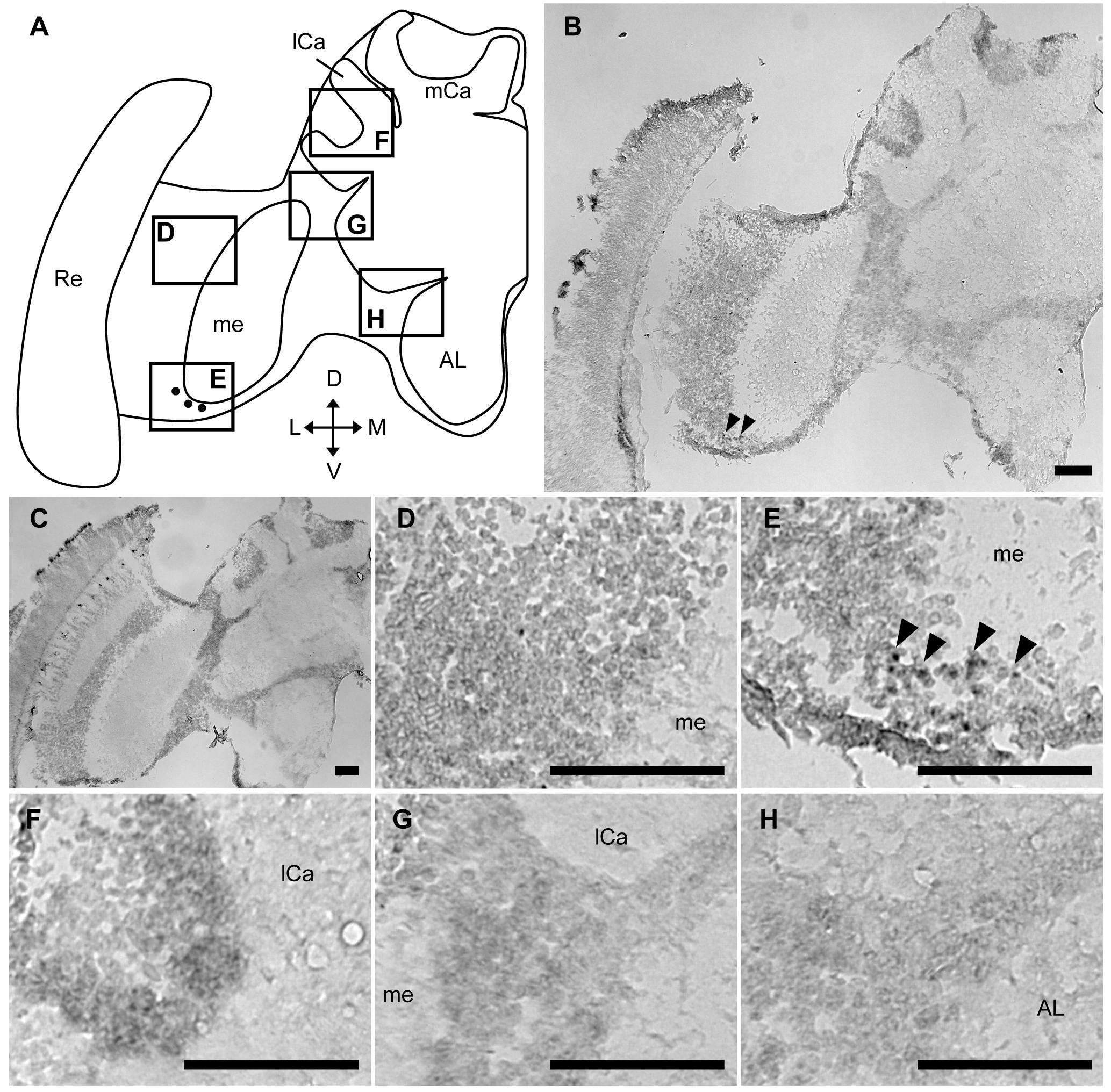

Supplement: Figure S8 — In situ hybridization with the intron probe of AmMESK2 in the nurse bee brains. In situ hybridization using DIG-labeled RNA antisense (B, D–H) and sense (C) AmMESK2 probes with nurse bee brain sections. (A) Schematic representation of signals detected in the left-brain hemisphere of the nurse bee brain. Black circles indicate stronger signals. (D–H) Magnified views of pars of (B) corresponding to the boxes shown in (A). The signals detected in the cortex between the lamina and medulla are indicated by black arrowheads. Scale bars = 100 µm. D, dorsal; L, lateral; AL, antennal lobe; lCa, lateral calyx; M, medial; me, medulla; mCa, medial calyx; Re, retina; V, ventral. (4.23 MB TIF) [file pone.0009213.s009.tif]

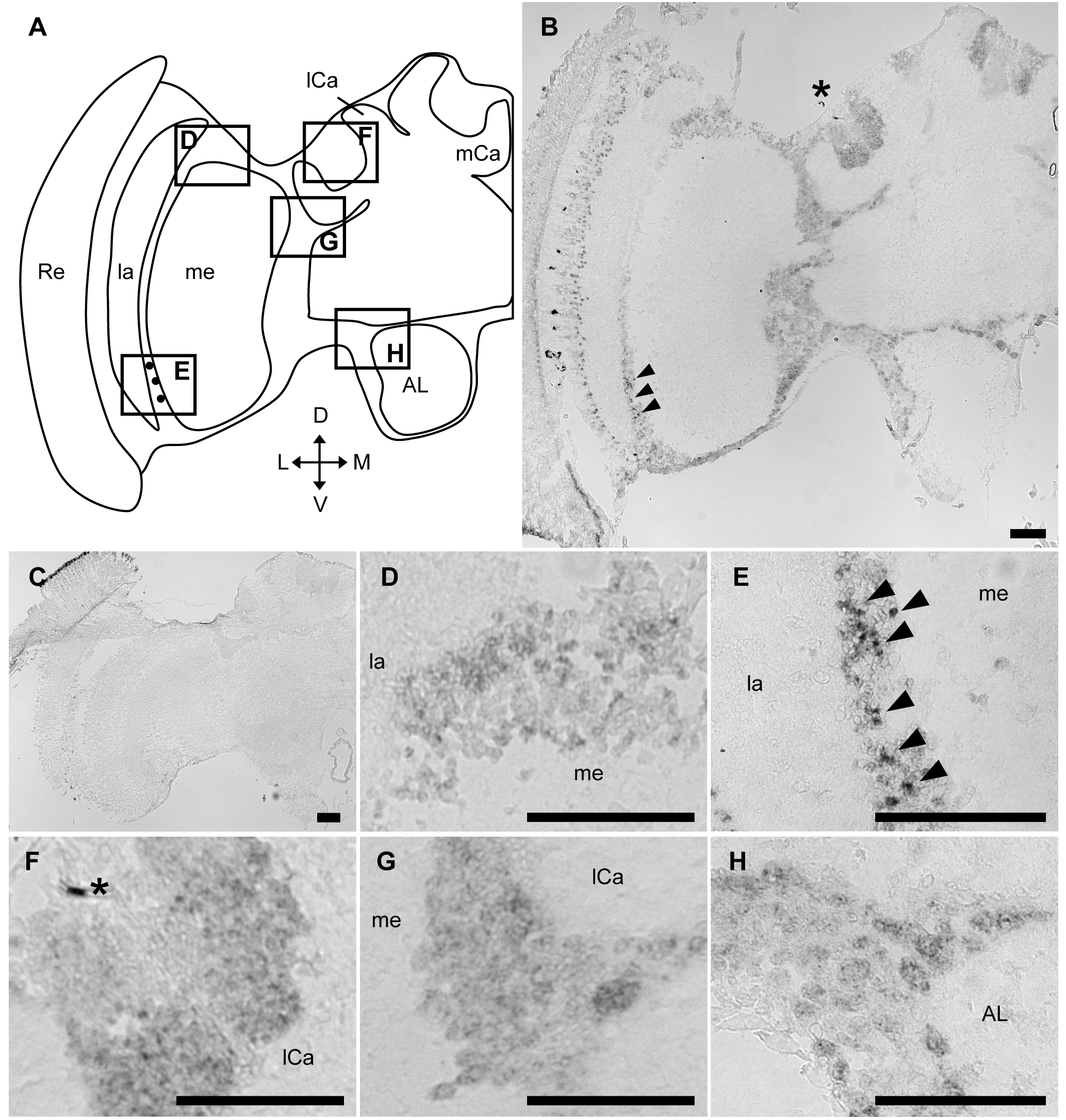

Supplement: Figure S9 — In situ hybridization with the exon probe of AmMESK2 in the nurse bee brains. In situ hybridization using DIG-labeled RNA antisense (B, D–H) and sense (C) AmMESK2 probes with nurse bee brain sections. (A) Schematic representation of signals detected in the left-brain hemisphere of the nurse bee brain. Black circles indicate stronger signals. (D–H) Magnified views of parts of (B) corresponding to the boxes shown in (A). The stronger signals detected between the lamina and medulla are indicated by black arrowheads. Scale bars = 100 µm. Asterisks indicate non-specific staining. AL, antennal lobe; D, dorsal; L, lateral; la, lamina; lCa, lateral calyx; M, medial; me, medulla; mCa, medial calyx; OL, optic lobe; Re, retina; V, ventral. (4.52 MB TIF) [file pone.0009213.s010.tif]

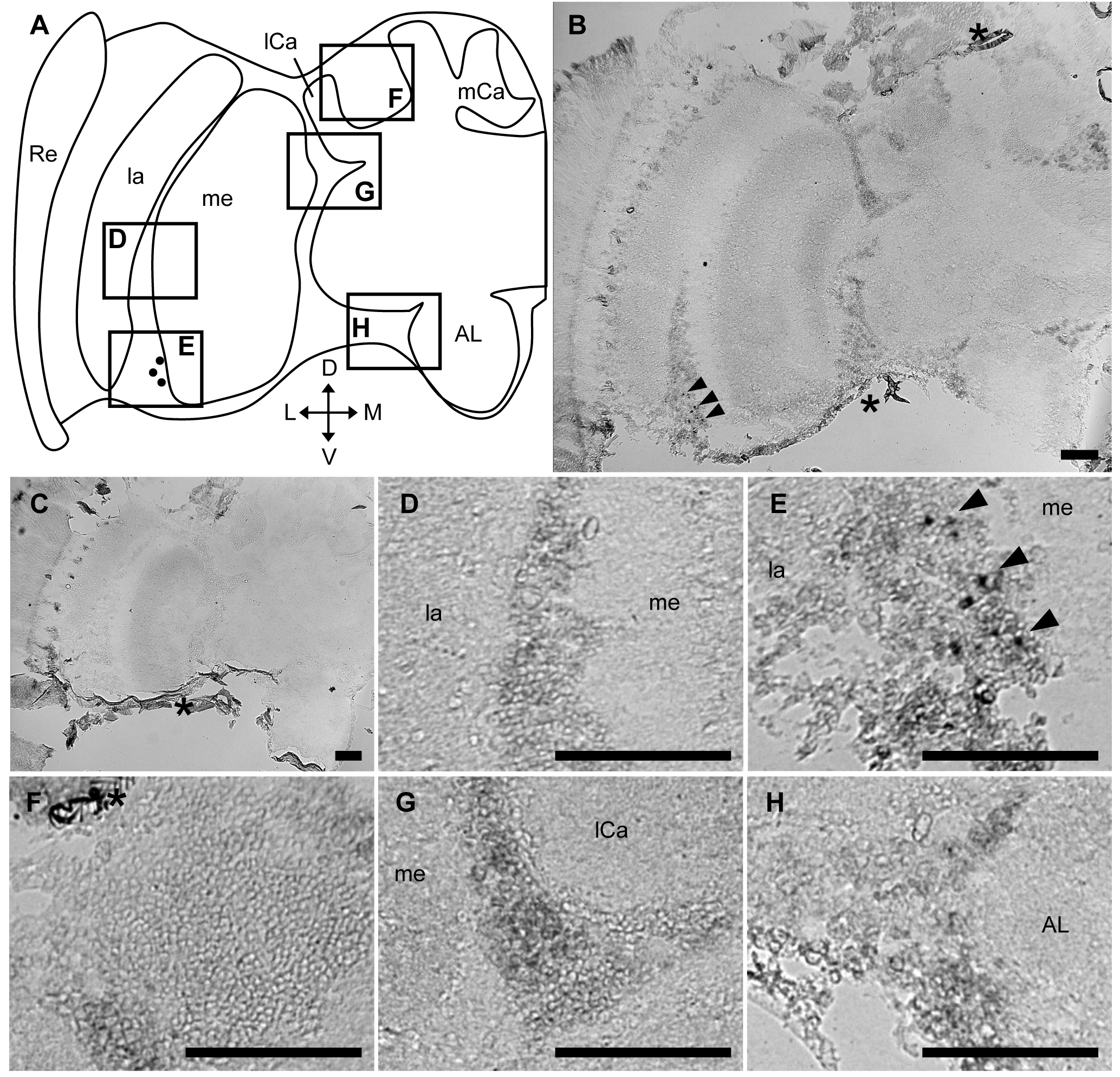

Supplement: Figure S10 — In situ hybridization with the exon probe of AmMESK2 in the queen brains. In situ hybridization using DIG-labeled RNA antisense (B, D–H) and sense (C) AmMESK2 probes with queen brain sections. (A) Schematic representation of signals detected in the left-brain hemisphere of the queen brain. Black circles indicate stronger signals. (D–H) Magnified views of parts of (B) corresponding to the boxes shown in (A). The stronger signals detected between the lamina and medulla are indicated by black arrowheads. Scale bars = 100 µm. Asterisks indicate non-specific staining. AL, antennal lobe; D, dorsal; L, lateral; la, lamina; lCa, lateral calyx; M, medial; me, medulla; mCa, medial calyx; OL, optic lobe; Re, retina; V, ventral. (4.14 MB TIF) [file pone.0009213.s011.tif]

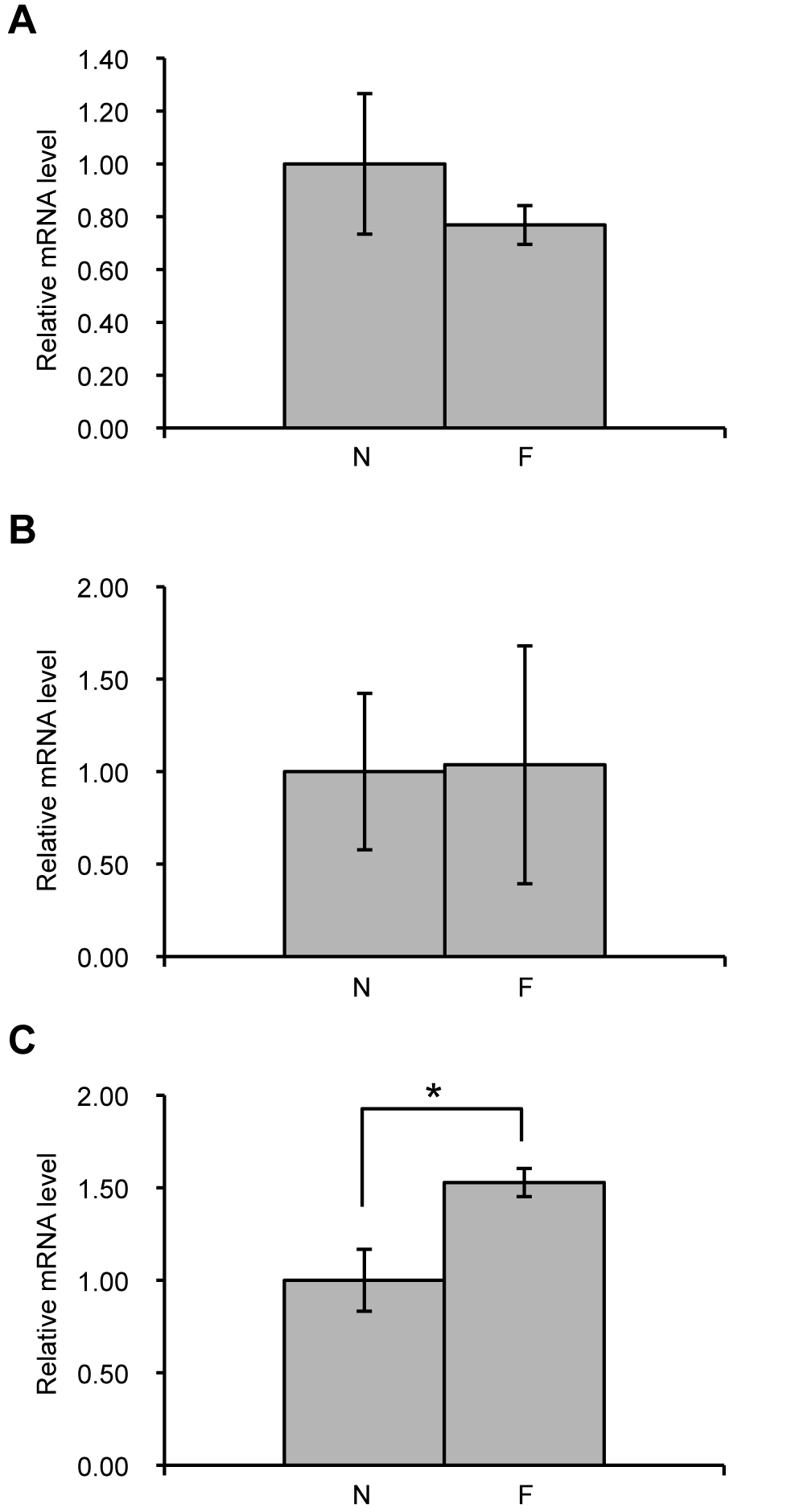

Supplement: Figure S11 — Comparison of the amounts of Amfutsch-, Amtau-, and AmMESK2-transcripts in the whole brains of queens, nurse bees, and foragers. Real-time RT-PCR was performed to compare the amounts of Amfutsch-, Amtau-, and AmMESK2-transcripts in the whole brains of nurse bees and foragers. N: nurse bee, F: forager. The amount of Amfutsch-, Amtau-, and AmMESK2-transcripts were normalized with that of actin defining the average of normalized mRNA levels in the nurse bee as 1. An asterisk indicates a significant difference between nurse bees and foragers (P<0.05; Welch's t-test). (1.90 MB TIF) [file pone.0009213.s012.tif]
